# Multiple aspects of amyloid dynamics *in vivo* integrate to establish prion variant dominance in yeast

**DOI:** 10.3389/fnmol.2024.1439442

**Published:** 2024-07-30

**Authors:** Jennifer Norton, Nicole Seah, Fabian Santiago, Suzanne S. Sindi, Tricia R. Serio

**Affiliations:** ^1^Department of Molecular and Cellular Biology, The University of Arizona, Tucson, AZ, United States; ^2^Department of Biochemistry, The University of Washington, Seattle, WA, United States; ^3^Department of Applied Mathematics, The University of California, Merced, Merced, CA, United States

**Keywords:** prion, amyloid, variant competition, chaperone, SUP35, Hsp104

## Abstract

Prion variants are self-perpetuating conformers of a single protein that assemble into amyloid fibers and confer unique phenotypic states. Multiple prion variants can arise, particularly in response to changing environments, and interact within an organism. These interactions are often competitive, with one variant establishing phenotypic dominance over the others. This dominance has been linked to the competition for non-prion state protein, which must be converted to the prion state via a nucleated polymerization mechanism. However, the intrinsic rates of conversion, determined by the conformation of the variant, cannot explain prion variant dominance, suggesting a more complex interaction. Using the yeast prion system [*PSI^+^*], we have determined the mechanism of dominance of the [*PSI^+^*]^Strong^ variant over the [*PSI^+^*]^Weak^ variant *in vivo*. When mixed by mating, phenotypic dominance is established in zygotes, but the two variants persist and co-exist in the lineage descended from this cell. [*PSI^+^*]^Strong^ propagons, the heritable unit, are amplified at the expense of [*PSI^+^*]^Weak^ propagons, through the efficient conversion of soluble Sup35 protein, as revealed by fluorescence photobleaching experiments employing variant-specific mutants of Sup35. This competition, however, is highly sensitive to the fragmentation of [*PSI^+^*]^Strong^ amyloid fibers, with even transient inhibition of the fragmentation catalyst Hsp104 promoting amplification of [*PSI^+^*]^Weak^ propagons. Reducing the number of [*PSI^+^*]^Strong^ propagons prior to mating, similarly promotes [*PSI^+^*]^Weak^ amplification and conversion of soluble Sup35, indicating that template number and conversion efficiency combine to determine dominance. Thus, prion variant dominance is not an absolute hierarchy but rather an outcome arising from the dynamic interplay between unique protein conformations and their interactions with distinct cellular proteostatic niches.

## 1 Introduction

Prions are proteins capable of transmitting novel physiological states, either vertically or horizontally, among individuals. In mammals, the archetype is the prion protein PrP, which is the infectious agent in the transmissible spongiform encephalopathies (TSEs), including Cruetzfeldt-Jakob’s disease (CJD) in humans, scrapie in sheep and goats, chronic wasting disease (CWD) in cervids, and bovine spongiform encephalopathy (BSE, also known as ‘mad-cow disease’) in cattle (McKinley et al., 1983; Kitamoto et al., 1986; Allsop et al., 1988; Hope et al., 1988; Borchelt et al., 1990; Caughey and Raymond, 1991; Bartz et al., 1998; Collinge, 2001). Prions have also been identified in fungi such as *S. cerevisiae* and *P. anserina* and are associated with the modulation of a wide range of cellular pathways including nitrogen metabolism and translation termination (Wickner, 2016). While these states are not considered to be pathogenic, they often alter the adaptive advantage of the organism (Tuite and Serio, 2010; Wickner, 2016). In all known systems, prion-associated physiological states are self-perpetuating, operationally expanding the function of genetic determination to proteins (Serio and Lindquist, 1999).

The engendering of genetic functions in prion proteins results directly from their conformational flexibility. These proteins can adopt alternative folds beyond their native structures that are rich in β-sheets. These secondary structures stack laterally through backbone hydrogen bonding to facilitate oligomerization and the formation of fibrillary structures known as amyloids (Pan et al., 1993; Chiti and Dobson, 2006; Wickner et al., 2008; Knowles et al., 2014), which also self-associate to form higher-order assemblies (Naeimi and Serio, 2022). Amyloidogenicity is frequently associated with intrinsically disordered regions of these proteins (Chiti and Dobson, 2016), resulting in a suite of amyloid structures for each protein (Diaz-Avalos et al., 2005; Nelson et al., 2005; Toyama et al., 2007). These quaternary structures are associated with distinct and transmissible biological outcomes, impacting the stability, severity, sequelae, incubation time, tropism, and interspecies transmissibility of the prion physiological state (Bessen and Marsh, 1994; Tanaka et al., 2006; Makarava et al., 2009; Terry et al., 2016). Together these structural and biological characteristics define unique prion variants, also known as strains, in mammals and fungi.

The variant-specific amyloid structures are defined by a core of distinct self-interactions among monomers of the same protein, which in turn determine the kinetic and thermodynamic stability of the complex and the minimum size of the assembly competent nucleation seed (Chiti et al., 2003; Knowles et al., 2014; Villali et al., 2020). These parameters connect the amyloid structure to its physiological impacts through an integrated system known as the nucleated polymerization model (NPM) (Nowak et al., 1998; Masel et al., 1999), which also captures the behavior of non-infectious amyloids such as α-synuclein in Parkinson’s Disease and Multiple System Atrophy (MSA) and Aβ and tau in Alzheimer’s disease, among others (Jucker and Walker, 2013; Walker et al., 2013). Specifically, each amyloid structure can be uniquely defined by three parameters: (1) the rate of conformational conversion of the non-prion conformer through association at and incorporation onto the fiber ends, (2) the rate of fragmentation of growing fibers to generate new templating surfaces, and the (3) the size-based persistence of individual aggregates arising from these dynamics (Masel et al., 1999; Tanaka et al., 2006; Villali et al., 2020). Together, these processes determine the variant-specific ratio of protein in the prion and non-prion states and the size distribution of amyloid aggregates *in vivo*. As a result, the degree of functional alteration of the protein is established and manifests as different “strengths” of the prion physiological state (Masel et al., 1999; Tanaka et al., 2006; Pezza et al., 2014). The transmissibility of the amyloid is also impacted by its size, contributing to the stability of the physiological state (Silveira et al., 2005; Derdowski et al., 2010).

Beyond existing as individual isolates, prion variants can co-exist within the same host. These mixtures may arise spontaneously (Collinge and Clarke, 2007; Bateman and Wickner, 2013). Evidence also suggests that existing variants can interchange, known as evolution, adaptation, or “mutation,” under selective pressure upon passage through a different host (Bruce and Dickinson, 1987; Santoso et al., 2000) or genetic background (Doel et al., 1994; Derkatch et al., 1999; DiSalvo et al., 2011; Lin et al., 2011; Yu and King, 2018) or in the presence of small molecules or pharmacological agents (Ghaemmaghami et al., 2009; Roberts et al., 2009; Shorter, 2010; Oelschlegel and Weissmann, 2013). At the phenotypic level, individual variants have been reported to establish dominance in mixtures, with the characteristics of the mixture typically being indistinguishable from those of the dominant variant (Tuite and Serio, 2010). Nonetheless, variant mixtures may also exhibit characteristics that are distinct from their isolated states (Kimberlin and Walker, 1978; Bessen and Marsh, 1992; Cali et al., 2009; Parchi et al., 2009). Thus, the interplay among prion variants is a crucial factor in understanding their physiological outcomes.

Studies of both mammalian and yeast prions suggest this interplay hinges on the relative rates of propagation of the interacting variants (Dickinson et al., 1972; Bradley et al., 2002; Tanaka et al., 2006). In the case of mammals, these differences have been shown to drive the competition for soluble prion protein, demonstrated *in vitro* in the presence of cellular cofactors using protein misfolding cyclic amplification (PMCA) (Atarashi et al., 2006, 2007; Bartz et al., 2007; Shikiya et al., 2010; Burke et al., 2020; Shikiya and Bartz, 2023). However, differences in the rate of conversion of non-prion state protein at the fiber ends cannot fully explain variant competition. Indeed, for the yeast prion [*PSI^+^*], the amyloid form of the Sup35 protein, the [*PSI^+^*]^Weak^ variant is recessive to the [*PSI^+^*]^Strong^ variant despite having a higher intrinsic rate of conformational conversion (Bradley et al., 2002; Tanaka et al., 2006). According to the NPM, the number of templates available also contributes to the rate of propagation (Nowak et al., 1998), and, consistent with this idea, variant competition is impacted by the interval between inoculations and by the relative titer of each variant in mammals and by the number of transmissible templates known as propagons in yeast (Dickinson et al., 1972; Dickinson and Outram, 1979; Manuelidis, 1998; Bartz et al., 2000, 2007; Cox et al., 2003; Manuelidis and Lu, 2003; Tanaka et al., 2006; Schutt and Bartz, 2008; Shikiya et al., 2010; Lin et al., 2011; Diaz-Espinoza et al., 2018; Shikiya and Bartz, 2023).

In yeast, the amplification of amyloid templates has been directly linked to the AAA+ ATPase Hsp104, which fragments amyloid fibers along with its co-chaperones Hsp70 (i.e., Ssa1/2), Hsp110 (i.e., Sse1/2) and Hsp40 (i.e., Sis1) (Chernoff et al., 1995; Song et al., 2005; Satpute-Krishnan et al., 2007; Higurashi et al., 2008; Sadlish et al., 2008; Tipton et al., 2008). A similar amyloid-fragmenting activity has been linked to the Hsp110, Hsp70, Hsp40 system in mammals (Gao et al., 2015). While factors other than the prion protein itself are involved in the formation of prion templates, the influence of this pathway on prion variant competition is mechanistically unexplored. Here, we assess this possibility using the yeast prion [*PSI^+^*], as a model system. We find that the [*PSI^+^*]^Strong^ and [*PSI^+^*]^Weak^ variants co-exist *in vivo* when introduced into the same cell by mating and compete directly for soluble Sup35 protein in the non-prion state. This competition is sensitive to the activity of Hsp104, which is necessary to amplify [*PSI^+^*]^Strong^ templates to a level capable of overcoming the higher rate of conversion of the [*PSI^+^*]^Weak^ variant. Thus, the integration of intrinsic and extrinsic forces shapes prion variant interactions.

## 2 Results

### 2.1 Prion variant dominance is established immediately upon mating

When [*PSI^+^*]^Strong^ and [*PSI^+^*]^Weak^ variants are introduced into the same cytoplasm by mating, the [*PSI^+^*]^Strong^ phenotype usually dominates at the colony level, following ~20 generations of growth (Derkatch et al., 1996; King, 2001; Bradley et al., 2002). However, it is unclear when or how this dominance is established. To examine prion variant competition in yeast, we first determined the temporal appearance of the [*PSI^+^*]^Strong^ phenotype during competition initiated by mating yeast haploids propagating different prion states and variants.

**Figure 1:**
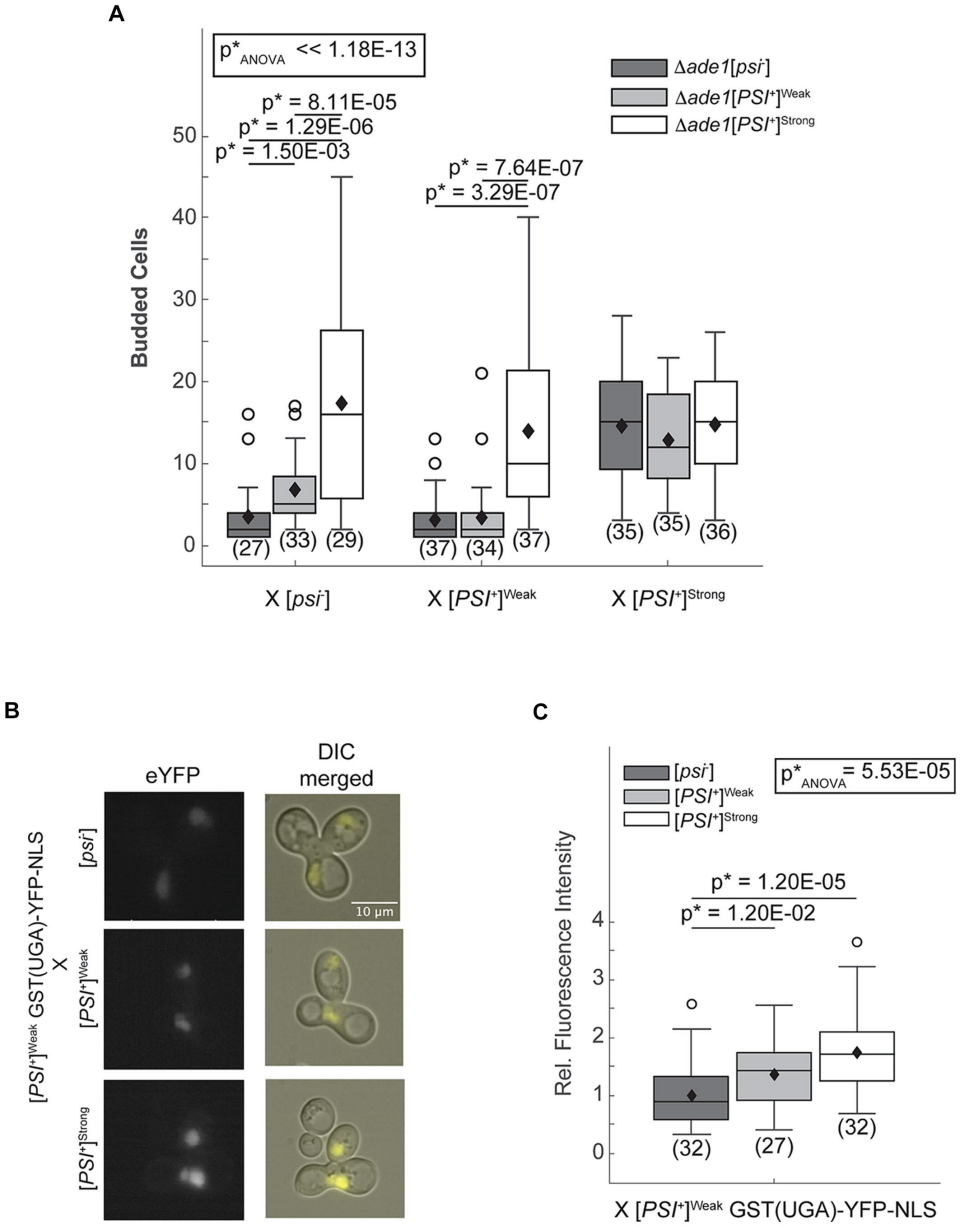
The [*PSI^+^*]^Strong^ phenotype is established immediately after variant mixing. **(A)** Δ*ade1* [*psi^−^*] (dark grey), Δ*ade1* [*PSI^+^*]^Weak^ (light gray), and Δ*ade1* [*PSI^+^*]^Strong^ (white) haploids were mated to wildtype (WT) [*psi^−^*], [*PSI^+^*]^Weak^, and [*PSI^+^*]^Strong^ haploids, and budding from the zygotes on synthetic complete medium was quantified. Results are presented as box-whisker plots. Horizontal lines indicate 25th, 50th, and 75th percentiles; whiskers indicate 10th and 90th percentiles; diamonds indicate means; outliers are presented as dots. The number of zygotes analyzed is presented in parentheses below each box plot. The equality of means across all groups was tested using Welch’s ANOVA test (p*_ANOVA_); *p*-values were determined using pairwise unequal variance *t-*tests and are presented above each box plot for comparisons within a cross. The absence of a *p*-value for comparisons within a cross indicates a lack of significant difference. Full statistical comparisons are available in Supplementary Table S1. **(B)** WT [*psi^−^*], [*PSI^+^*]^Strong^, and [*PSI^+^*]^Weak^ haploids were mated to a [*PSI^+^*]^Weak^ haploid expressing a fluorescent, nuclear localizing readthrough reporter, GST(UGA)-YFP-NLS. Representative epifluorescence images (eYFP) of the zygotes are shown alongside merged eYFP and Normarski images (DIC). **(C)** Fluorescence intensity within zygotes, representing stop codon readthrough levels, was quantified and presented in a box-whisker plot. Horizontal lines indicate 25th, 50th, and 75th percentiles; whiskers indicate 10th and 90th percentiles; diamonds indicate means; outliers are presented as dots. The number of zygotes analyzed is presented in parentheses below each box plot. The equality of means across all groups was tested using Welch’s ANOVA test (p*_ANOVA_); *p*-values were determined using pairwise unequal variance *t-*tests and are presented above each box plot for comparisons within a cross. The absence of a *p*-value for comparisons within a cross indicates a lack of significant difference. Full statistical comparisons are available in Supplementary Table S2.

The [*PSI^+^*] prion-associated phenotype can be monitored at the colony level in strains with the *ade1-14* genotype, which encodes a premature stop codon in the *ADE1* open reading frame (Chernoff et al., 1995; Fitzpatrick et al., 2011). These strains can only grow in the absence of exogenous adenine if the premature stop codon is read-through efficiently as occurs in [*PSI^+^*]^Strong^ but not [*PSI^+^*]^Weak^ or non-prion [*psi^−^*] strains (Chernoff et al., 1995; Derkatch et al., 1996). To remove the interference of existing Ade1 protein in the crosses, we used one haploid that is disrupted for *ADE1* (*Δade1*) in each cross, with the *ade1-14* allele encoded in the other mating partner. As expected, zygotes resulting from mating an *ade1-14* [*psi^−^*] haploid and an *Δade1* [*psi^−^*] or [*PSI^+^*]^Weak^ haploid divide only a few times when transferred to medium lacking adenine (Figure 1A, dark and light gray, respectively); however, zygotes resulting from mating an *ade1-14* [*psi^−^*] haploid and an *Δade1* [*PSI^+^*]^Strong^ haploid divide robustly in the absence of adenine (Figure 1A, white). Because the *ade1-14* reporter only encounters the [*PSI^+^*]^Strong^ state upon mating, the prion-associated readthrough phenotype must be established in the zygote.

To determine whether the [*PSI^+^*]^Strong^ phenotype is established on the same timescale upon mating [*PSI^+^*]^Strong^ and [*PSI^+^*]^Weak^ haploids, we also created [*PSI^+^*]^Strong^ (Figure 1A, white) and [*PSI^+^*]^Weak^ (Figure 1A, light gray) strains with *ADE1* disruptions. Zygotes formed by mating these haploids with an *ade1-14* [*psi^−^*] haploid divide similarly to those formed by mating them with an *ade1-14* haploid propagating the same prion variant (i.e., mating two [*PSI^+^*]^Strong^ or two [*PSI^+^*]^Weak^ haploids; Figure 1A). In these crosses, the [*PSI^+^*]^Strong^ zygotes divide more robustly than the [*PSI^+^*]^Weak^ zygotes in the absence of adenine as expected. Thus, the prion-associated phenotypes of [*PSI^+^*]^Strong^ and [*PSI^+^*]^Weak^ are preserved in this experimental system. Zygotes formed by mating an *ade1-14* [*PSI^+^*]^Weak^ haploid and an *Δade1* [*PSI^+^*]^Strong^ haploid divide robustly in the absence of adenine and indistinguishably from a zygote formed by mating *ade1-14* and *Δade1* [*PSI^+^*]^Strong^ haploids together (Figure 1A, white), indicating a similar phenotype. Because the *ade1-14* [*PSI^+^*]^Weak^ haploid is unable to divide robustly in the absence of adenine when mated to either an *Δade1* [*psi^−^*] or an [*PSI^+^*]^Weak^ haploid (Figure 1A, dark and light gray, respectively), our observations indicate that the [*PSI^+^*]^Strong^ variant establishes phenotypic dominance over the [*PSI^+^*]^Weak^ variant in the zygote.

As an alternative and more sensitive approach to assess the emergence of the [*PSI^+^*]^Strong^ phenotype in zygotes, we directly monitored stop codon readthrough efficiency using a YFP-based fluorescent reporter [i.e., GST(UGA)-YFP-NLS], whose nuclear expression requires the readthrough of a stop codon (Langlois et al., 2016). Zygotes formed by mating a [*psi^−^*] haploid expressing the reporter to [*psi^−^*], [*PSI^+^*]^Weak^, or [*PSI^+^*]^Strong^ haploids exhibit increasing nuclear fluorescence intensities, respectively (Supplementary Figure S1). Thus, the reporter captures the phenotypic differences in the efficiency of stop codon readthrough expected of these strains (Derkatch et al., 1996). We next expressed the reporter in a [*PSI^+^*]^Weak^ haploid and performed the same crosses. Upon mating to a [*psi^−^*] haploid, weak fluorescence is observed in the zygote. This baseline level of fluorescence increases in zygotes formed by mating a [*PSI^+^*]^Weak^ haploid expressing the reporter to a [*PSI^+^*]^Weak^ and to a [*PSI^+^*]^Strong^ haploid (Figures 1B,C). Because this level of stop codon readthrough exceeds that observed in the zygotes formed by mating the reporter haploid to a [*psi^−^*] haploid (Figures 1B,C), [*PSI^+^*]^Strong^ has established phenotypic dominance in the zygote.

### 2.2 [*PSI^+^*]^Strong^ phenotypic dominance is established without elimination of [*PSI^+^*]^Weak^ aggregates

**Figure 2:**
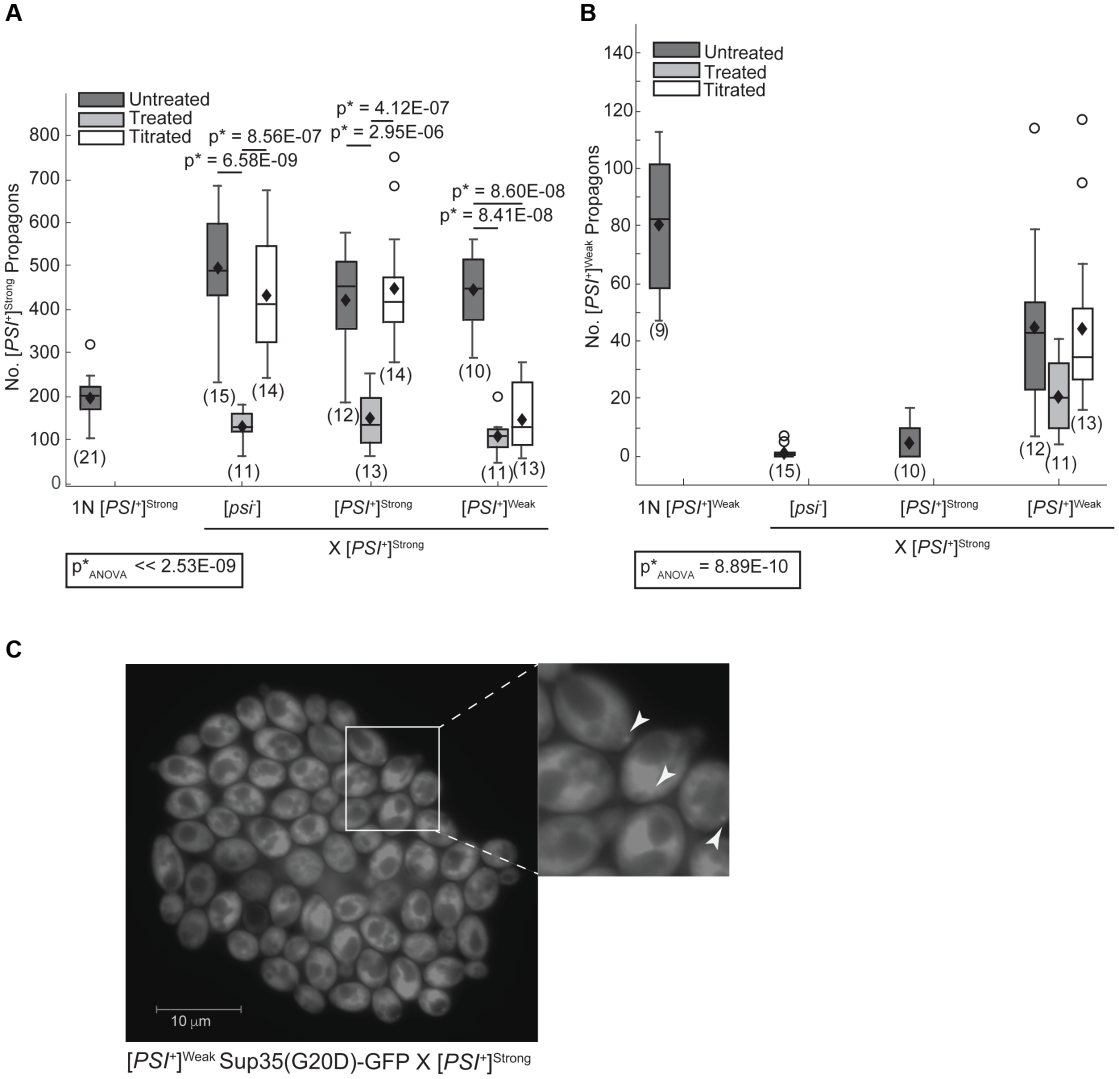
The [*PSI^+^*]^Weak^ variant persists after mating to the dominant [*PSI^+^*]^Strong^ variant. **(A)** Wildtype (WT) [*psi^−^*], [*PSI^+^*]^Weak^, and [*PSI^+^*]^Strong^ haploids were mated to a WT [*PSI^+^*]^Strong^ haploid without treatment (untreated, dark gray), in the presence of 3 mM GdnHCl (treated, light gray), and in the presence of 3 mM GdnHCl following growth of the [*PSI^+^*]^Strong^ haploid (1N) in the presence of GdnHCl for 12 h (titrated, white). The number of [*PSI^+^*]^Strong^ propagons from the resulting zygotes and a [*PSI^+^*]^Strong^ haploid was quantified and presented as box-whisker plots. Horizontal lines indicate 25th, 50th, and 75th percentiles; whiskers indicate 10th and 90th percentiles; diamonds indicate means; outliers are presented as dots. The number of zygotes analyzed is presented in parentheses below each box plot. The equality of means across all groups was tested using Welch’s ANOVA test (p*_ANOVA_); *p*-values were determined using pairwise unequal variance *t-*tests and are presented above each box plot for comparisons within a cross. The absence of a *p*-value for comparisons within a cross indicates a lack of significant difference. Full statistical comparisons are available in Supplementary Table S3. **(B)** The number of [*PSI^+^*]^Weak^ propagons from the zygotes presented in **(A)** was quantified and presented as box-whisker plots as in **(A)**. The equality of means across all groups was tested using Welch’s ANOVA test (p*_ANOVA_); *p*-values were determined using pairwise unequal variance *t-*tests and are presented above each box plot for comparisons within a cross. The absence of a *p*-value for comparisons within a cross indicates a lack of significant difference. Full statistical comparisons are available in Supplementary Table S4. **(C)** The persistence of the [*PSI^+^*]^Weak^ variant was observed by microscopy in crosses between [*PSI^+^*]^Strong^ and [*PSI^+^*]^Weak^ haploids expressing Sup35(G20D)-GFP under the control of the P*_tetO7_* promoter. GFP foci were detected in cells at the periphery of the resulting microcolony, indicated by white arrows in the inset (200-fold magnification).

The [*PSI^+^*]^Strong^ variant may establish phenotypic dominance by eliminating Sup35 aggregates of the [*PSI^+^*]^Weak^ variant or through other changes in Sup35 biogenesis. To directly assess whether Sup35 aggregates of the [*PSI^+^*]^Weak^ variant persist in zygotes after mating [*PSI^+^*]^Strong^ and [*PSI^+^*]^Weak^ haploids, we quantified variant-specific heritable prion aggregates, known as propagons, in zygotes using an established plating assay (Cox et al., 2003). When a [*PSI^+^*]^Strong^ haploid is mated to [*psi*^−^], [*PSI^+^*]^Strong^, and [*PSI^+^*]^Weak^ haploids, [*PSI^+^*]^Strong^ propagons are recovered from the zygotes (Figure 2A, dark gray). Minimal and indistinguishable levels of [*PSI^+^*]^Weak^ propagons are recovered from zygotes formed by mating a [*PSI^+^*]^Strong^ haploid to [*psi*^−^] or [*PSI^+^*]^Strong^ haploids (Figure 2B, dark gray) However, a significant number of [*PSI^+^*]^Weak^ propagons are recovered from the zygote fromed by mating [*PSI^+^*]^Strong^ and [*PSI^+^*]^Weak^ haploids (Figure 2B, dark gray). Thus, [*PSI^+^*]^Weak^ propagons, deposited into the zygote by mating, persist at least initially in the presence of [*PSI^+^*]^Strong^.

To determine whether Sup35^[*PSI+*]Weak^ aggregates persist as the zygote formed by mating [*PSI^+^*]^Strong^ and [*PSI^+^*]^Weak^ haploids divides to form a microcolony, we monitored their persistence using a GFP fusion to the G20D mutant of Sup35 (King, 2001), which forms fluorescent foci in [*PSI^+^*]^Weak^ but not in [*PSI^+^*]^Strong^ strains (Supplementary Figure S2A). As a control, we mated a [*psi^−^*] haploid expressing a tetracycline-inducible copy of Sup35(G20D)-GFP to [*psi^−^*], [*PSI^+^*]^Strong^, and [*PSI^+^*]^Weak^ haploids and then induced expression of the GFP-tagged protein following the transfer of zygotes to medium containing doxycycline. In the resulting microcolonies, Sup35(G20D)-GFP remains diffuse when the reporter strain is mated to either [*psi^−^*] (Supplementary Figure S2B) or [*PSI^+^*]^Strong^ haploids (Supplementary Figure S2C), but fluorescent foci are visible in some descendants of zygotes resulting from crosses to a [*PSI^+^*]^Weak^ haploid (Supplementary Figure S2D), demonstrating the specificity of this approach.

To assess the persistence of [*PSI^+^*]^Weak^ aggregates in the presence of [*PSI^+^*]^Strong^ on this timescale, we next mated a [*PSI^+^*]^Weak^ haploid encoding the tetracycline-inducible Sup35(G20D)-GFP reporter to a [*PSI^+^*]^Strong^ haploid. Following transfer of zygotes to medium containing doxycycline, GFP-labeled foci were observed at the periphery of the microcolony (Figure 2C). Using confocal microscopy, foci were visible in all cells in the microcolony, paralleling observations when the same reporter haploid strain is mated to a [*psi^−^*] haploid (Supplementary Figures S2E,F). The detection of Sup35(G20D)-GFP foci further demonstrates that Sup35 aggregates of the [*PSI^+^*]^Weak^ variant are not eliminated during competition with [*PSI^+^*]^Strong^ upon mating and persist at least for several generations.

### 2.3 [*PSI^+^*]^Strong^ interferes with the amplification of [*PSI^+^*]^Weak^ propagons but not *vice-versa*

Given the persistence of Sup35^[*PSI+*]Weak^ aggregates in the presence of [*PSI^+^*]^Strong^ (Figure 2), prion variant competition likely arises through an alteration in the biogenesis of Sup35 in the presence of [*PSI^+^*]^Strong^. To explore the potential targets of such an alteration, we first assessed propagon numbers in zygotes formed by mixing different prion states and variants.

In zygotes formed by mating a [*PSI^+^*]^Strong^ haploid to a [*psi^−^*] or [*PSI^+^*]^Strong^ haploid, [*PSI^+^*]^Strong^ propagons accumulate to similar levels (Figure 2A, dark gray). This level is approximately 2.5-fold higher than the propagons present in a [*PSI^+^*]^Strong^ haploid (Figure 2A, 1N); thus [*PSI^+^*]^Strong^ propagons are rapidly amplified in the zygote. Notably, zygotes formed by mating [*PSI^+^*]^Strong^ and [*PSI^+^*]^Weak^ haploids accumulate a similar level of [*PSI^+^*]^Strong^ propagons to the former zygotes (Figure 2A, compare dark gray); thus, the presence of [*PSI^+^*]^Weak^ does not interfere with the amplification of [*PSI^+^*]^Strong^ propagons in zygotes.

**Figure 3:**
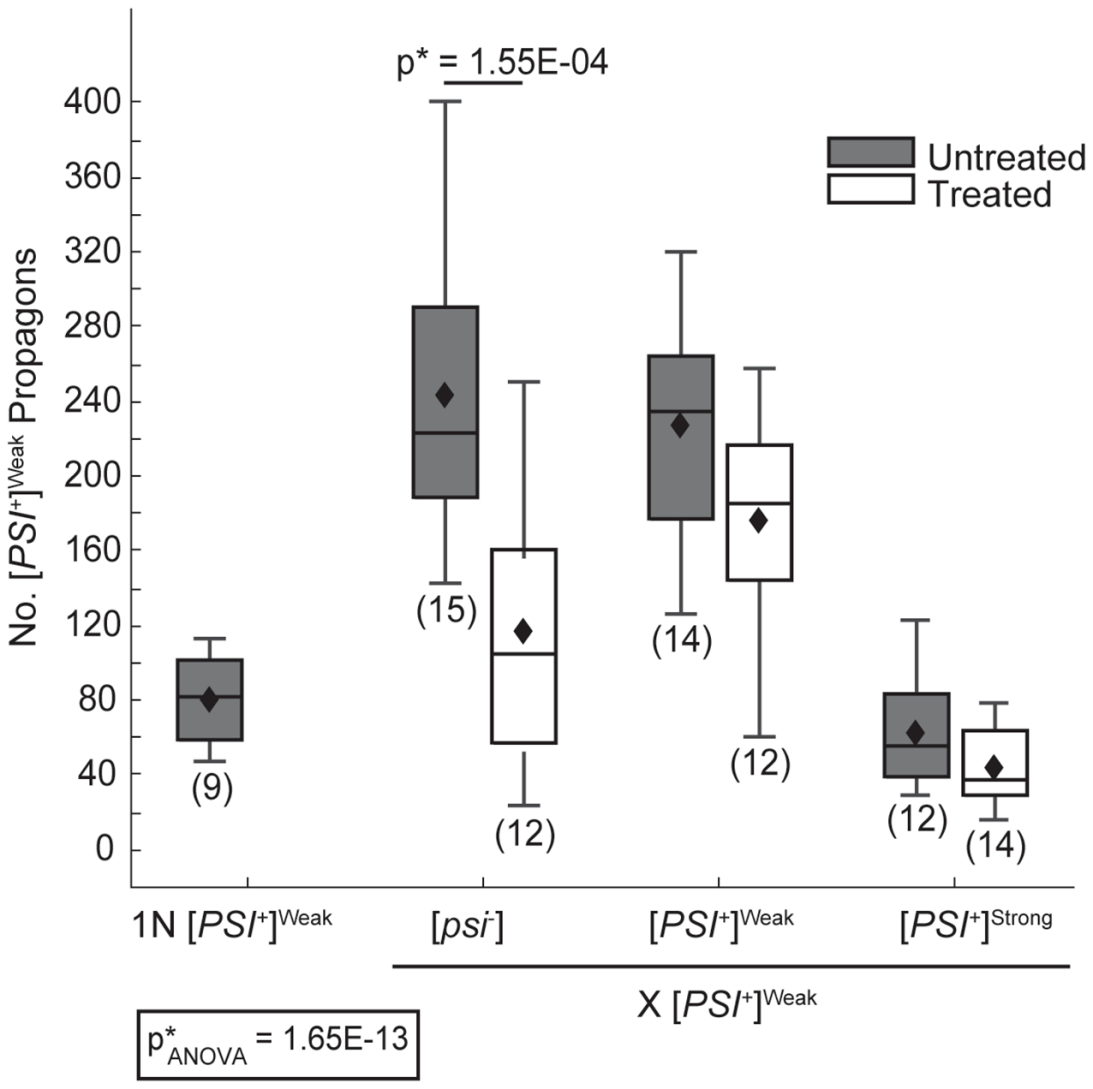
The dominant [*PSI^+^*]^Strong^ variant interferes with amplification of [*PSI^+^*]^Weak^ propagons. A wildtype (WT) [*PSI^+^*]^Weak^ haploid (1 N) was mated to WT [*psi^−^*], [*PSI^+^*]^Weak^, and [*PSI^+^*]^Strong^ haploids without treatment (untreated, dark gray) or in the presence of 3 mM GdnHCl (treated, white). The number of [*PSI^+^*]^Weak^ propagons from the zygotes and the [*PSI^+^*]^Weak^ haploid was quantified and presented in box-whisker plots. Horizontal lines indicate 25th, 50th, and 75th percentiles; whiskers indicate 10th and 90th percentiles; diamonds indicate means. The number of zygotes analyzed is presented in parentheses below each box plot. The equality of means across all groups was tested using Welch’s ANOVA test (p*_ANOVA_); *p*-values were determined using pairwise unequal variance *t-*tests and are presented above each box plot for comparisons within a cross; the absence of a *p*-value for comparisons within a cross indicates a lack of significant. Full statistical comparisons are available in Supplementary Table S5.

In zygotes formed by mating a [*PSI^+^*]^Weak^ haploid to [*psi^−^*] or [*PSI^+^*]^Weak^ haploids, [*PSI^+^*]^Weak^ propagons accumulate to a similar level (Figure 3, dark gray), and again, this level is approximately 2.5-fold higher than that observed for a [*PSI^+^*]^Weak^ haploid (Figure 3, 1N). Thus, [*PSI^+^*]^Weak^ propagons also amplify rapidly in the zygote. In contrast, zygotes formed by mating [*PSI^+^*]^Weak^ and [*PSI^+^*]^Strong^ haploids accumulate [*PSI^+^*]^Weak^ propagons at levels that are similar to the haploid (Figure 3, dark gray); thus, the presence of [*PSI^+^*]^Strong^ interferes with the amplification of [*PSI^+^*]^Weak^ propagons in these zygotes. Because this interference impacts the amplification of [*PSI^+^*]^Weak^ (Figure 3) but not [*PSI^+^*]^Strong^ (Figure 2A) propagons and is aligned with the phenotypic dominance of the latter (Figure 1), variant competition occurs at the level of amplification of the prion heritable unit.

### 2.4 [*PSI^+^*]^Strong^ outcompetes [*PSI^+^*]^Weak^ for conversion of Sup35 *in vivo*

Amplification of propagons requires conversion of soluble Sup35 to the prion state and fragmentation and transmission of prion aggregates; thus, competition could arise at either or both of these events. Both empirical data and mathematical models propose that conversion of soluble protein to the prion state is key to determining outcomes in prion variant competition (Tanaka et al., 2006; Shikiya et al., 2010). Given the phenotypic dominance of [*PSI^+^*]^Strong^, we predict that soluble Sup35 will be more efficiently converted to the [*PSI^+^*]^Strong^ than the [*PSI^+^*]^Weak^ state in zygotes propagating both variants. To directly test this idea, we monitored Sup35-GFP transmissibility by fluorescence loss in photobleaching (FLIP), which we have previously demonstrated to be a sensitive assay of Sup35 physical state (Derdowski et al., 2010).

**Figure 4:**
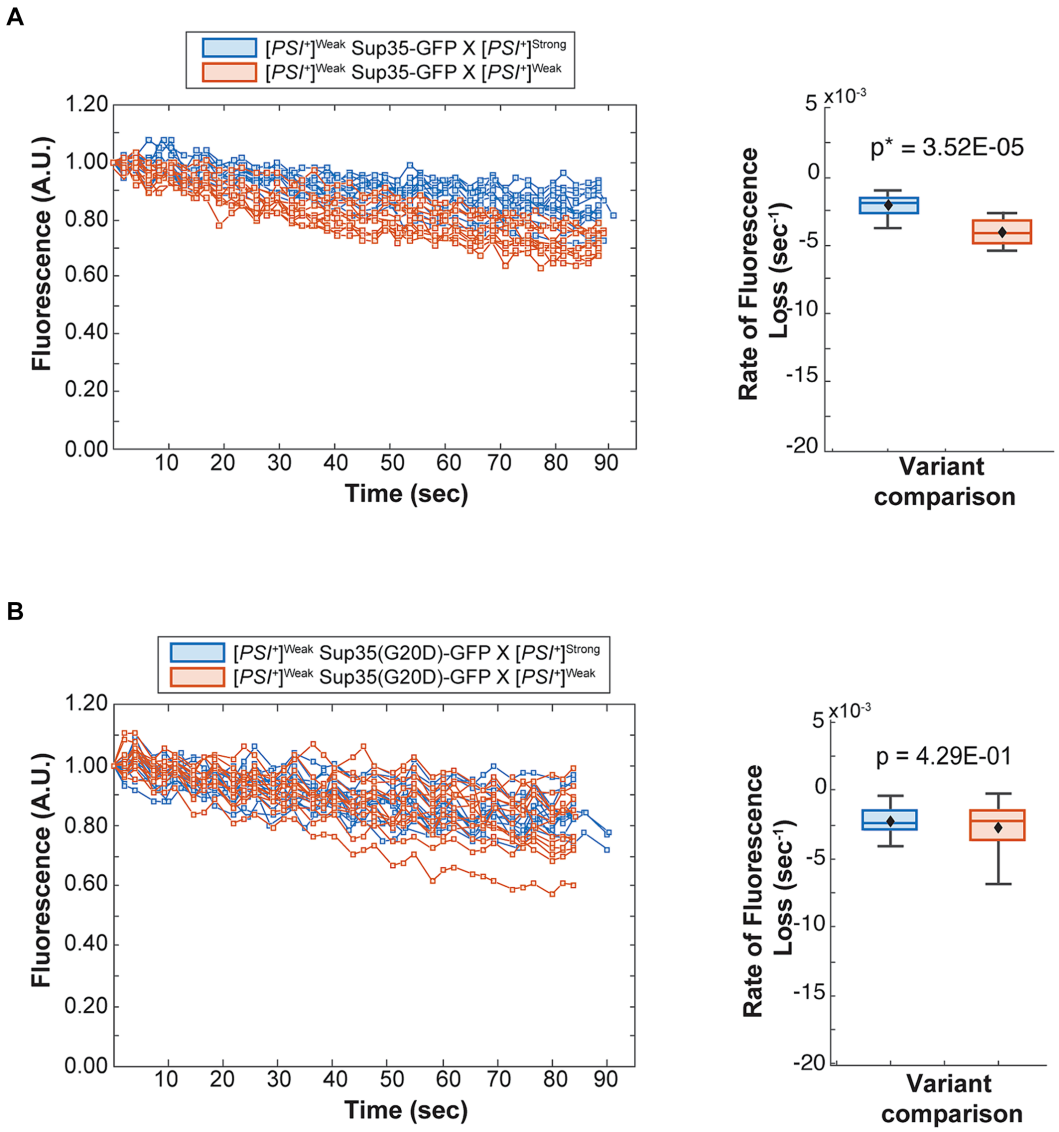
The dominant [*PSI^+^*]^Strong^ variant competes for soluble Sup35 more efficiently than the recessive [*PSI^+^*]^Weak^ variant. **(A)** Wildtype [*PSI^+^*]^Strong^ (blue) and [*PSI^+^*]^Weak^ haploids (salmon) were mated to a [*PSI^+^*]^Weak^ haploid expressing GFP-tagged Sup35 under the control of the P*_MFA1_* promoter. Fluorescence intensity was quantified in the mother lobe of a singly budded zygote following repeated photobleaching of the daughter (left), and the rate of fluorescence loss was calculated and presented as box-whisker plots (right, *n* ≥ 11). Horizontal lines indicate 25th, 50th, and 75th percentiles; whiskers indicate 10th and 90th percentiles; diamonds indicate means. An unequal variance *t*-test was used to determine *p* values **(B)** A [*PSI^+^*]^Weak^ haploid expressing a GFP-tagged Sup35 mutant (G20D) under the control of the P*_MFA1_* promoter was mated to wildtype [*PSI^+^*]^Strong^ (blue) and [*PSI^+^*]^Weak^ (salmon) haploids and analyzed as in (**A**, *n* ≥ 13).

To determine the fate of Sup35 protein derived from a [*PSI^+^*]^Weak^ haploid in zygotes, we mated a [*PSI^+^*]^Weak^ haploid expressing Sup35-GFP to [*PSI^+^*]^Weak^ or [*PSI^+^*]^Strong^ haploids and monitored fluorescence loss in the dumbbell-shaped fused haploids during bleaching of the daughter bud. In matings between [*PSI^+^*]^Weak^ haploids, fluorescence is lost at a rate of 4.2 × 10^−3^ s^−1^ (Figure 4A, salmon and Table 1). In matings between [*PSI^+^*]^Weak^ and [*PSI^+^*]^Strong^ haploids, fluorescence is lost at a rate of 2.3 × 10^−3^ s^−1^ (Figure 4A, blue and Table 1). The reduced rate of fluorescence loss across the bud neck in the zygotes formed by mating [*PSI^+^*]^Strong^ and [*PSI^+^*]^Weak^ haploids is consistent with an increase in aggregated Sup35 relative to that found in zygotes formed by mating [*PSI^+^*]^Weak^ haploids alone. Because this decreased rate correlated with the amplification of [*PSI^+^*]^Strong^ propagons (Figure 2A), it likely represents the incorporation of soluble Sup35 into aggregates in the [*PSI^+^*]^Strong^ state.

**Table 1:** Fluorescence loss in photobleaching.

**Cross**	**Average (sec** ^**−1**^ **)**	**95% CI (sec** ^**−1**^ **)**
[*PSI^+^*]^Strong-GFP^ × [*PSI^+^*]^Strong^	−0.00438	(−5.73 × 10^−3^, −3.04 × 10^−3^)
[*PSI^+^*]^Strong-GFP^ × [*PSI^+^*]^Weak^	−0.01258	(−1.44 × 10^−2^, −1.08 × 10^−2^)
[*PSI^+^*]^Strong(11–61)-GFP^ × [*PSI^+^*]^Strong^	−0.00904	(−1.03 × 10^−2^, −7.74 × 10^−3^)
[*PSI^+^*]^Strong(11–61)-GFP^ × [*PSI^+^*]^Weak^	−0.00786	(−9.05 × 10^−3^, −6.67 × 10^−3^)
[*PSI^+^*]^Weak-GFP^ × [*PSI^+^*]^Strong^	−0.00225	(−2.78 × 10^−3^, −1.73 × 10^−3^)
[*PSI^+^*]^Weak-GFP^ × [*PSI^+^*]^Weak^	−0.00424	(−4.88 × 10^−3^, −3.59 × 10^−3^)
[*PSI^+^*]^Weak(G20D)-GFP^ × [*PSI^+^*]^Strong^	−0.00242	(−3.03 × 10^−3^, −1.81 × 10^−3^)
[*PSI^+^*]^Weak(G20D)-GFP^ × [*PSI^+^*]^Weak^	−0.00288	(−3.95 × 10^−3^, −1.81 × 10^–3)^

If this prediction is accurate, the reduction in the rate of fluorescence loss in the presence of [*PSI^+^*]^Strong^ should not be observed in strains expressing Sup35(G20D)-GFP, which cannot be converted to the [*PSI^+^*]^Strong^ state (Supplementary Figure S2A) (King, 2001). To test this idea, we mated a [*PSI^+^*]^Weak^ haploid expressing Sup35(G20D)-GFP to [*PSI^+^*]^Weak^ and [*PSI^+^*]^Strong^ haploids. Fluorescence is lost at a rate of 2.9 × 10^−3^ s^−1^ in the zygotes formed by mating the [*PSI^+^*]^Weak^ haploids alone and at a rate of 2.4 × 10^−3^ s^−1^ in the zygotes formed by mating the [*PSI^+^*]^Strong^ and [*PSI^+^*]^Weak^ haploids (Figure 4B, salmon and blue, respectively, and Table 1), consistent with our prediction. Thus, the amplification of [*PSI^+^*]^Strong^ propagons and its phenotypic dominance in the presence of [*PSI^+^*]^Weak^ in zygotes are associated with the efficient incorporation of Sup35 into aggregates of the [*PSI^+^*] ^Strong^ variant, providing direct evidence of competition at the protein level.

### 2.5 Amplification of [*PSI^+^*] prion variants is differentially sensitive to Hsp104 activity

While our observations provide direct evidence of prion variant competition at the level of conversion of soluble Sup35 protein, the mechanism through which this competition arises remains unclear. For example, the preferential incorporation of Sup35 into aggregates of the [*PSI^+^*]^Strong^ variant in the presence of aggregates of the [*PSI^+^*]^Weak^ variant (Figure 4) cannot be explained by their biochemical properties, as the former catalyzes conversion at a lower rate than the latter *in vitro* (Tanaka et al., 2006). Thus, additional aspects of the *in vivo* prion cycle must impact variant competition.

Mathematical models of prion propagation indicate that the conversion of soluble protein is determined by the inherent rate of conversion by aggregates as well as by their number, which is tightly linked to the rate of fragmentation (Nowak et al., 1998; Masel et al., 1999). Although [*PSI^+^*]^Weak^ propagons are present in zygotes formed by mating a [*PSI^+^*]^Strong^ and a [*PSI^+^*]^Weak^ haploid (Figure 2B), they are not amplified to the diploid level (Figure 3, dark gray), perhaps suggesting a reduced rate of fragmentation during competition. To assess the fragmentation efficiency of aggregates of the [*PSI^+^*]^Weak^ variant in the presence or absence of aggregates of the [*PSI^+^*]^Strong^ variant, we introduced a copy of Sup35 tagged with GFP that is expressed from the P*_MFA1_* promoter. The P*_MFA1_* promoter is a mating type **a**-specific promoter that is repressed upon mating (Galgoczy et al., 2004); thus, Sup35-GFP synthesis ceases, and the fate of the existing protein can be monitored over time (Satpute-Krishnan et al., 2007). Previously, we demonstrated that [*PSI^+^*]^Strong^ cells expressing Sup35-GFP from P*_MFA1_* gradually lose their fluorescent foci due to fragmentation by Hsp104 and the incorporation of unlabeled Sup35, which continues to be synthesized (Satpute-Krishnan et al., 2007). However, if the fragmentation activity of Hsp104 is inhibited with guanidine HCl (GdnHCl) (Ferreira et al., 2001; Jung and Masison, 2001; Grimminger et al., 2004), GFP foci remain visible (Satpute-Krishnan et al., 2007). If Sup35-GFP aggregates of the [*PSI^+^*]^Weak^ variant are poorly fragmented in the presence of the [*PSI^+^*]^Strong^ variant, we would expect the fluorescent foci to persist longer than in the absence of the competing prion.

**Figure 5:**
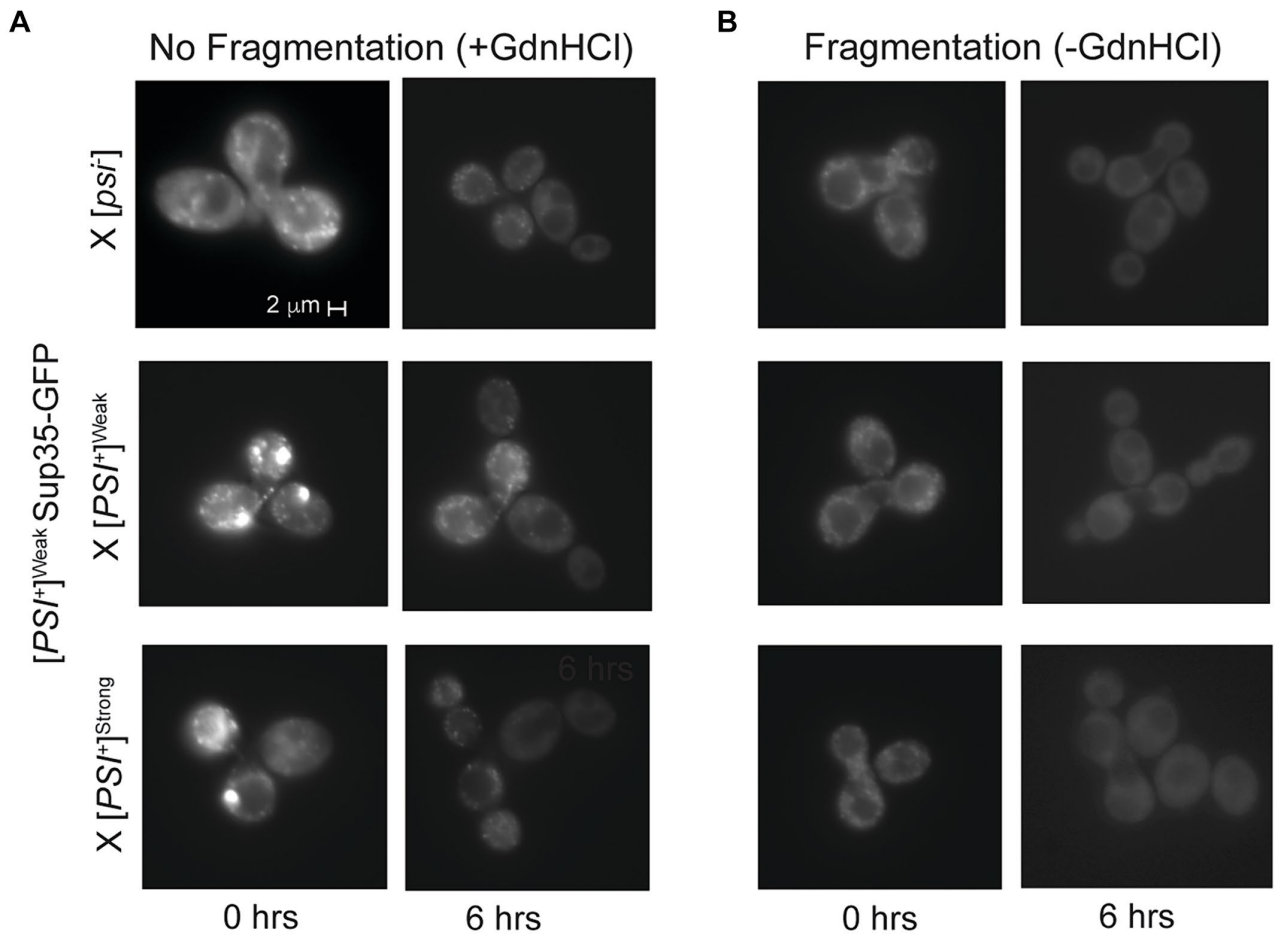
Competing variants are differentially affected by fragmentation activity. **(A,B)** A [*PSI^+^*]^Weak^ haploid expressing GFP-tagged Sup35 under the control of the P*_MFA1_* promoter was mated to the indicated wildtype haploids in the presence or absence of guanidine hydrochloride (+/-GdnHCl). For each cross, a representative zygote (left column) and its resulting microcolony (right column) are shown (*n* > 8). Note that the differences in fluorescent intensity between zygotes (+/- GdnHCl) are explained by the incorporation of soluble Sup35-GFP onto newly introduced prion aggregates that are or are not being fragmented, as expected (Satpute-Krishnan et al., 2007).

Upon mating a [*PSI^+^*]^Weak^ haploid expressing Sup35-GFP from P*_MFA1_* to [*psi*^−^], [*PSI^+^*]^Weak^, or [*PSI^+^*]^Strong^ haploids, the resulting zygotes contain several GFP-labeled foci (Figure 5A), and these complexes persist over several hours if fragmentation is inhibited with GdnHCl (Figure 5A, right panel). In the absence of GdnHCl, fluorescent foci are no longer visible by 6 h in the zygotes formed by mating a [*PSI^+^*]^Weak^ haploid expressing Sup35-GFP from P*_MFA1_* to [*psi*^−^] and [*PSI^+^*]^Weak^ haploids, indicating that fragmentation of these aggregates is robust (Figure 5B, right panel). Similarly, fluorescent foci are lost by 6 h in the absence of GdnHCl in zygotes formed by mating a [*PSI^+^*]^Weak^ haploid expressing Sup35-GFP from P*_MFA1_* to a [*PSI^+^*]^Strong^ haploid, suggesting the presence of the [*PSI^+^*]^Strong^ variant does not substantially interfere with the fragmentation of Sup35 aggregates of the [*PSI^+^*]^Weak^ variant (Figure 5B, right panel). Thus, prion variant competition is unlikely to arise through a modulation of fragmentation rate.

While the fragmentation of Sup35 aggregates of the [*PSI^+^*]^Weak^ variant is not disadvantaged by the presence of the [*PSI^+^*]^Strong^ variant, fragmentation could still provide an advantage to the latter by promoting the observed amplification of propagons (Figure 2A). To determine how fragmentation rates affect the amplification of Sup35 propagons during mating, we assessed their number in the presence or absence of GdnHCl treatment. When a [*PSI^+^*]^Strong^ haploid is mated to [*psi*^−^], [*PSI^+^*]^Weak^ and [*PSI^+^*]^Strong^ haploids in the presence of GdnHCl, there is a significant drop in the number of [*PSI^+^*]^Strong^ propagons relative to the untreated crosses (Figure 2A, compare dark and light gray). In contrast, there is no significant change in the number of [*PSI^+^*]^Weak^ propagons in the zygotes formed by mating [*PSI^+^*]^Strong^ and [*PSI^+^*]^Weak^ haploids in presence or absence of GdnHCl (Figure 2B, compare dark and light gray). This differential impact of fragmentation on [*PSI^+^*]^Strong^ and [*PSI^+^*]^Weak^ propagons results in a change in their ratio from 9:1 in the untreated zygote to 5:1 in the presence of GdnHCl (Figures 2A,B). Thus, the amplification of [*PSI^+^*]^Strong^ propagons is more sensitive to the fragmentation activity of Hsp104 than that of [*PSI^+^*]^Weak^ propagons.

### 2.6 Template number drives prion variant competition *in vivo*

**Figure 6:**
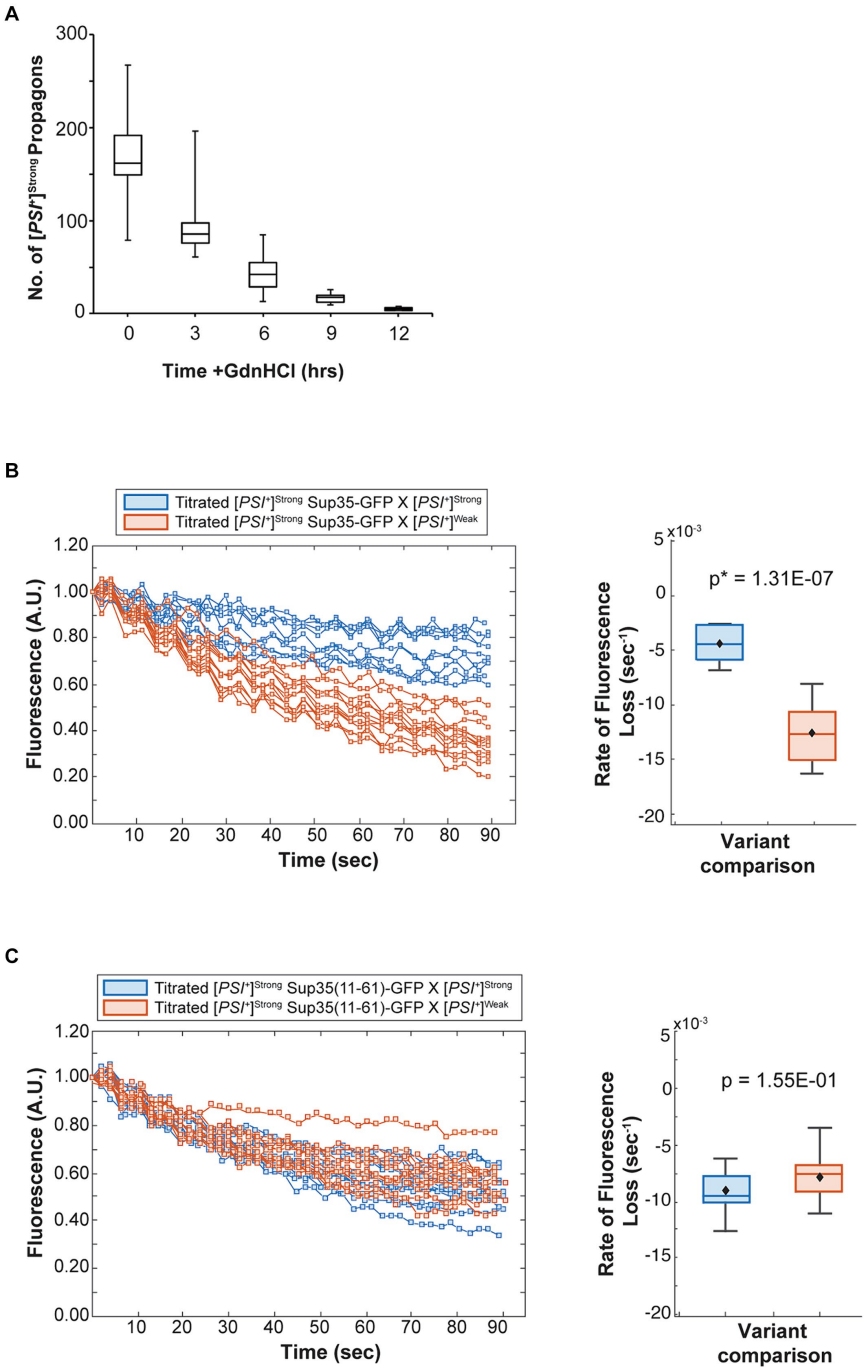
The competitive advantage of [*PSI^+^*]^Strong^ is limited by template abundance. **(A)** The number of [*PSI*^+^]^Strong^ propagons was determined at 3-h intervals in the presence of guanidine hydrochloride (GdnHCl) (*n* > 12). Results are presented as box-whisker plots. Horizontal lines indicate 25th, 50th, and 75th percentiles; whickers indicate 10th and 90th percentiles. **(B)** A [*PSI^+^*]^Strong^ haploid expressing GFP-tagged Sup35 under the control of the P*_MFA1_* promoter was grown in the presence of GdnHCl for 12 h then mated to wildtype [*PSI^+^*]^Strong^ (blue) or [*PSI^+^*]^Weak^ haploids (salmon). The daughters of singly budded zygotes were subjected to repeated photobleaching while fluorescence intensity in the mother lobes was monitored (left) and the rates of fluorescence decay were calculated and presented as box-whisker plots (right, *n* ≥ 9). Horizontal lines indicate 25th, 50th, and 75th percentiles; whiskers indicate 10th and 90th percentiles. An unequal variance *t*-test was used to determine *p*-values. **(C)** A [*PSI^+^*]^Strong^ haploid expressing a GFP-tagged Sup35 mutant (11–61) under the control of the P*_MFA1_* promoter was grown in the presence of GdnHCl for 12 h and then crossed to wildtype [*PSI^+^*]^Strong^ (blue) and [*PSI^+^*]^Weak^ (salmon) haploids and analyzed as in (**B**, *n* ≥ 11).

While the efficient incorporation of Sup35 protein (Figure 4 and Table 1) and the phenotypic dominance (Figure 1) of the [*PSI^+^*]^Strong^ variant in the presence of the [*PSI^+^*]^Weak^ variant cannot be explained by its lower inherent rate of conversion, the relative sensitivity of the amplification of [*PSI^+^*]^Strong^ propagons to Hsp104 activity may suggest that template number is a key determinant of variant competition. To test this idea, we grew a [*PSI^+^*]^Strong^ haploid in the presence of GdnHCl for 12 h, hereafter referred to as titrated [*PSI^+^*]^Strong^. This treatment leads to a reduction in [*PSI^+^*]^Strong^ propagons (Figure 6A) to a level below that of [*PSI^+^*]^Weak^ propagons in [*PSI^+^*]^Weak^ haploids (Figure 3) without curing [*PSI^+^*]^Strong^. Consistent with the decrease in propagons, the titrated [*PSI^+^*]^Strong^ haploid has a corresponding increase in soluble Sup35, similar to the level found in a [*psi^−^*] haploid (Supplementary Figure S3).

To determine how the number of [*PSI^+^*]^Strong^ propagons affects competition, we mated the titrated [*PSI^+^*]^Strong^ haploid with [*psi^−^*], [*PSI^+^*]^Strong^, and [*PSI^+^*]^Weak^ haploids in the presence of GdnHCl. The amplification of [*PSI^+^*]^Strong^ propagons observed in zygotes formed by mating a [*PSI^+^*]^Strong^ haploid to [*psi*^−^] and [*PSI^+^*]^Strong^ haploids is also observed when a titrated [*PSI^+^*]^Strong^ haploid is mated to the same haploids (Figure 2A, compare dark gray to white), indicating rapid amplification of propagons in the zygotes. Paralleling our observations in zygotes formed in the presence of GdnHCl (Figure 2A, light gray), the amplification of [*PSI^+^*]^Strong^ propagons in zygotes formed by mating a titrated [*PSI^+^*]^Strong^ strain to a [*PSI^+^*]^Weak^ strain remains inhibited (Figure 2A, compare light gray to white). This observation suggests that [*PSI^+^*]^Weak^ can effectively interfere with the amplification of [*PSI^+^*]^Strong^ propagons when the propagon levels of the two variants are more balanced.

Despite this inhibition of amplification of [*PSI^+^*]^Strong^ propagons, we also do not observe amplification of [*PSI^+^*]^Weak^ propagons, which accumulate to indistinguishable levels in the zygotes formed by mating a [*PSI^+^*]^Weak^ haploid to either a titrated [*PSI^+^*]^Strong^ or [*PSI^+^*]^Strong^ haploid in the presence of GdnHCl (Figure 2B, compare light gray to white). Because the amplification of [*PSI^+^*]^Strong^ propagons correlates with the conversion of Sup35 to the [*PSI^+^*]^Strong^ state (Figures 2A, 4), we hypothesized that [*PSI^+^*]^Weak^ more effectively competes with [*PSI^+^*]^Strong^ for Sup35 when propagon levels are more balanced. If this were the case, amplification of [*PSI^+^*]^Weak^ propagons is unlikely to be observed due to the incorporation of soluble Sup35 onto the larger aggregates present in a [*PSI^+^*]^Weak^ strain, which would have reduced transmission (Derdowski et al., 2010). Indeed, the amplification of [*PSI^+^*]^Weak^ propagons is significantly reduced in the presence of GdnHCl when a [*PSI^+^*]^Weak^ haploid is mated to a [*psi^−^*] haploid but not to [*PSI^+^*]^Weak^ or [*PSI^+^*]^Strong^ haploids (Figure 3, compare white), the former of which contains high levels of soluble Sup35 as is the case for titrated [*PSI^+^*]^Strong^ (Supplementary Figure S3).

To test this idea, we monitored the fate of Sup35 protein by FLIP in zygotes formed by mating a titrated [*PSI^+^*]^Strong^ strain expressing Sup35-GFP to either a [*PSI^+^*]^Strong^ or [*PSI^+^*]^Weak^ strain. Fluorescence is lost at a rate of 4.4 × 10^−3^ s^−1^ in the zygotes formed by mating titrated [*PSI^+^*]^Strong^ and [*PSI^+^*]^Strong^ haploids (Figure 6B, blue and Table 1), but the rate of fluorescence loss is more rapid (1.3 × 10^−2^ s^−1^) when the same strain is mated to a [*PSI^+^*]^Weak^ haploid (Figure 6B, salmon and Table 1). These observations indicate that, when [*PSI^+^*]^Strong^ propagons are limiting, Sup35-GFP is more efficiently converted to the [*PSI^+^*] state (i.e., slower rate of fluorescence loss) in the absence of the [*PSI^+^*]^Weak^ variant, consistent with the interference of [*PSI^+^*]^Weak^ in the amplification of [*PSI^+^*]^Strong^ propagons (Figure 2A).

If this interpretation is accurate, [*PSI^+^*]^Weak^ should not interfere with the conversion of a Sup35 mutant that can only adopt the [*PSI^+^*]^Strong^ state. To test this idea, we used the Sup35(11–61) fragment, which, when fluorescently tagged, forms foci in [*PSI^+^*]^Strong^ but not [*PSI^+^*]^Weak^ strains (Supplementary Figure S2) as previously suggested (King, 2001). We expressed Sup35(11–61)-GFP in the titrated [*PSI^+^*]^Strong^ strain and monitored its fate by FLIP in zygotes formed by mating to either [*PSI^+^*]^Strong^ or [*PSI^+^*]^Weak^ haploids. Fluorescence is lost at a rate of 9 × 10^−3^ s^−1^ in zygotes formed by mating to a [*PSI^+^*]^Strong^ haploid and at a rate of 7.9 × 10^−3^ s^−1^ in zygotes formed by mating to a [*PSI^+^*]^Weak^ haploid (Figure 6C, blue and salmon, respectively, and Table 1). The similar rates of fluorescence loss for the Sup35(11–61)-GFP protein in these two zygotes contrasts with the differential rates of fluorescence loss for wildtype Sup35-GFP in similar zygotes (Figure 6B and Table 1) and indicates that [*PSI^+^*]^Weak^ competes directly with [*PSI^+^*]^Strong^ for the conversion of Sup35 when propagons of the latter are limiting.

## 3 Discussion

Observations of interference among prion variants pre-date the prion hypothesis (Dickinson et al., 1972), but within this framework, the prevailing model links variant dominance to the competition for soluble prion protein (Bradley et al., 2002; Tanaka et al., 2006; Shikiya et al., 2010; Eckland et al., 2018). This conclusion is inferred from prion interference studies *in vitro*, using recombinant PrP^C^ as the substrate in a simplified PMCA system (Atarashi et al., 2007). Here, our studies using the yeast [*PSI^+^*] prion system provide direct *in vivo* evidence of variant competition for soluble Sup35 prion protein. Specifically, soluble Sup35 is efficiently converted to the dominant [*PSI^+^*]^Strong^ state in the presence of the recessive [*PSI^+^*]^Weak^ prion (Figure 4).

This observation, while aligned with *in vitro* studies in mammals, cannot be explained by the intrinsic rate of conversion of the variants (Tanaka et al., 2006) nor can it account for transitions among prion variants from established mixtures, where dominance has already been established. However, our studies provide a new pathway for understanding these dynamics by probing early events following the mixing of prion variants in individual cells. When [*PSI^+^*]^Weak^ and [*PSI^+^*]^Strong^ variants are mixed in zygotes, the transmissible unit (i.e., the propagon) of the dominant [*PSI^+^*]^Strong^ variant is immediately amplified (Figure 2A), and this amplification is associated with an inhibition in amplification of propagons of the recessive [*PSI^+^*]^Weak^ variant (Figure 3). Within the context of the NPM, such amplification is based on rates of both conversion and fragmentation (Nowak et al., 1998; Masel et al., 1999). Previous studies have estimated the product of those rates to be higher for the dominant [*PSI^+^*]^Strong^ variant, despite its reduced rate of conversion at fiber ends, in comparison with the recessive [*PSI^+^*]^Weak^ variant (Tanaka et al., 2006), suggesting an enhanced dependence on fragmentation for the former. Indeed, transiently blocking the fragmentation activity of Hsp104 more significantly interferes with the amplification of [*PSI^+^*]^Strong^ than [*PSI^+^*]^Weak^ propagons in zygotes (Figures 2A,B); in contrast, the incorporation of soluble Sup35 more significantly interferes with the amplification of [*PSI^+^*]^Weak^ than [*PSI^+^*]^Strong^ propagons (Figures 2A,B). Together, these observations suggest, for the first time, that the interplay between both amyloid conversion and fragmentation are key factors in the competition among prion variants.

This expanded view of competition among prion variants provides a direct explanation for experimental manipulations known to alter the dominance of prion variants in mammals. Specifically, a recessive variant can effectively compete with a normally dominant variant if the former has increased titer or is inoculated in advance (Dickinson et al., 1972; Bartz et al., 2000). Presumably, the additional templates of the recessive variant resulting from these conditions are sufficient to overcome its relatively inefficient amplification, providing competitive advantage to the otherwise recessive variant. Indeed, the amplification of a recessive variant is necessary for efficient competition in mammals (Kimberlin and Walker, 1985; Bartz et al., 2007; Shikiya et al., 2010). As a direct test of this prediction, our studies demonstrate that the recessive [*PSI^+^*]^Weak^ variant effectively competes for soluble Sup35 protein when the titer of the normally dominant [*PSI^+^*]^Strong^ variant is reduced by treatment with GdnHCl (Figure 6). This advantage, however, is transient, as colonies formed from diploid strains resulting from the mating of the titrated [*PSI^+^*]^Strong^ haploid to [*psi^−^*], [*PSI^+^*]^Weak^, and [*PSI^+^*]^Strong^ haploids all have the [*PSI^+^*]^Strong^ phenotype (Supplementary Figure S4), indicating that the dominant variant can overcome this initial setback.

The invariant dominance of [*PSI^+^*]^Strong^, even when titrated, that we observe differs from a previous report, in which [*PSI^+^*]^Weak^ emerged in approximately one third of the progeny of diploids formed by mating [*PSI^+^*]^Strong^ and [*PSI^+^*]^Weak^ haploids (Sharma and Liebman, 2012). While we have been unable to reproduce this result with either our own or the original yeast strains, we cannot rule out the possibility that the original [*PSI^+^*]^Weak^ variant has evolved over time. Nonetheless, our observations that the [*PSI^+^*]^Weak^ variant persists in these diploids (Figure 2) and is capable of incorporating soluble Sup35 (Figure 4) and being processed by Hsp104 (Figure 5) suggest that variant competition is an on-going process in cells containing variant mixtures. As such, it can be altered, leading to re-emergence of a recessive variant if more favorable conditions arise.

The co-existence of yeast prion variants in individual cells is consistent with the co-existence of PrP^Sc^ variants in mammals, including in humans (Polymenidou et al., 2005; Collinge and Clarke, 2007; Cassard et al., 2020). Such mixtures are particularly relevant for the transmission of prions among species and during exposure to anti-prion therapeutics, two conditions in which variant switches are often observed (Bruce and Dickinson, 1987; Ghaemmaghami et al., 2009; Roberts et al., 2009; Li et al., 2010; Oelschlegel and Weissmann, 2013). Although it is unclear whether these new variants are a result of conformational “mutation” or whether they are selected from an existing mixture, the transition, at some point, must involve the co-existence of distinct variants and a change in their competitive advantage. Such dynamics are difficult to reconcile with a model based solely on the intrinsic conversion rates of the corresponding amyloid structures. Rather, insight into these transitions must consider the broader system in which the competition for soluble prion protein occurs. The protein quality control apparatus that senses and responds to proteotoxic stress is a particularly relevant component of this system, given its known impact on prion aggregate fragmentation in yeast (Chernoff et al., 1995; Song et al., 2005; Satpute-Krishnan et al., 2007; Higurashi et al., 2008; Sadlish et al., 2008; Tipton et al., 2008). Indeed, mutations in chaperone proteins have previously been reported to differentially impact prion variants (Astor et al., 2018; Yu and King, 2018). Thus, the sensitivity of prion variant competition to even transient changes in chaperone activity in yeast that we have observed here provides an expanded framework for addressing the seemingly expansive adaptability of prion variants *in vivo*.

## 4 Materials and methods

### 4.1 Plasmid construction

All bacterial plasmids used in this study are listed in Table 2. All oligonucleotides are listed in Table 3.

**Table 2:** Bacterial plasmids.

**Name**	**Description**	**Reference**
SB869	pRS303P*_MFA1_Sup35(G20D)GFP*	This study
SB1047	pRS306P*_tetO7_Sup35(G20D)GFP*	This study
SB661	pRS303P_*tetO7*R’_	This study
SB1120	pRS303P*_MFA1_Sup35(11-61)*mCherry	This study
SB117	pRS303P*_MFA1_Sup35GFP*	Satpute-Krishnan and Serio (2005)
SB105	pRS303P*_MFA1_*	Satpute-Krishnan and Serio (2005)
SB604	pRS304P*_MFA1_N(GS)3mCherry(GS)*	Satpute-Krishnan and Serio (2005)
SB1133	pRS303P*_MFA1_Sup35(11-61)GFP*	This study
SB910	pRS304P*_GPD_GST(UGA)YFPNLS*	Langlois et al. (2016)

**Table 3:** Oligonucleotides.

**Name**	**Sequence**
ADE13’KO	5’ GAGGAGTTACACTGGCGACTTGTAGTATATGTAAATCACGGCATAGGCCACTAGTGGATCTG 3’
ADE15’KO	5’ CATTGCTTACAAAGAATACACATACGAAATATTAACGATACAGCTGAAGCTTCGTACGC 3’
Ade15’Ch	5’ CTTACCAAGCAGAGAATGTT 3’
Ade13’Ch	5’ AATGTGACACCGTCCCTG 3’
G20D Sup35 F	5’ CAGCAATACAGCCAGAACGATAACCAACAACAAGGTAAC 3’
G20D Sup35 R	5’ GTTACCTTGTTGTTGGTTATCGTTCTGGCTGTATTGCTG 3’
G20D check	5’ GCTACGGTTGGCCCATACCTTTAT 3’
F 11–61 BamHI	5’ AAAGGATCCATGCAAAACTACCAGCAATACAGCC 3’
R 11–61 XbaI	5’ AAATCTAGATTGATAGCCACCTTGTTGGTACCC 3’
F mcherry XbaI	5’AAATCTAGAGGTAGTGGTAGTGGTAGTATGGTGAGCAAGGGC 3’
R mcherry SacI	5’AAAGAGCTCTTACTTGTACAGCTCGTCCATGCCGGCC 3’
F GFP XbaI	5’ AAATCTAGAATGGCTAGCAAAGGAGAAGAACTCTTCACTGG 3’
R GFP SacI	5’ AAAGAGCTCTTATTTGTATAGTTCATCCATGCCATGTGTAATCCC 3’

SB869 was constructed from SB117 using QuikChangeII site-directed mutagenesis kit (Agilent) with primers G20DSup35 F and G20DSup35 R. The mutation was confirmed by sequencing using G20D check. SB1047 was constructed by subcloning the tetracycline inducible promoter, P*_tetO7_*, from SB661 into SB869 via restriction sites *ClaI* and *BamHI*. SB1120 was constructed by amplifying the coding sequence for methionine plus amino acids 11–61 from SB117 using primers F 11–61 BamHI and R 11–61 Xba1, followed by subcloning into SB105. The mCherry sequence was amplified from SB604 using primers XbaI mcherry F and SacI mcherry R and subcloned into SB101. SB1133 was constructed by amplifying GFP coding sequence from SB117 with primers F GFP XbaI and R GFP SacI. The amplified sequence was and subcloned into SB1120 via *XbaI* and *SacI* restriction sites. All restriction enzymes were obtained from New England BioLabs, Ipswitch, MA.

### 4.2 Strain construction

All yeast strains used in this study are derivatives of the 74-D694 strain (Chernoff et al., 1995) and are listed in Table 4. All oligonucleotides are listed in Table 3.

**Table 4:** *Saccharomyces cerevisiae* strains.

**Strain**	**Genotype**	**Plasmids integrated**	**Reference**	**Figures**
SLL2606	*MATa* [*PSI^+^*]*^Strong^ ade1-14 his3Δ200 trp1–289 ura3-52 leu2-3, 112*	–	Chernoff et al. (1995)	Figures 1B,C, 2A, 6 and Supplementary Figures S1, S3
SLL2119	*MATa* [*psi^−^*] *ade1-14 his3Δ200 trp1-289 ura3-52 leu2-3, 112*	–	Chernoff et al. (1995)	Figures 1B,C and Supplementary Figures S1, S3
SLL2600	*MATa* [*PSI^+^*]*^Weak^ ade1-14 his3Δ200 trp1-289 ura3-52 leu2-3, 112*	–	Derkatch et al. (1996)	Figures 1B,C, 3 and Supplementary Figure S1
SY3609	*MATα* [*PSI^+^*]*^Weak^ ade1-14 his3Δ200 trp1-289 ura3-52 leu2-3, 112*	–	This study	
SLL3250	*MATα* [*PSI^+^*]*^Strong^ ade1-14 his3Δ200 trp1–289 ura3-52 leu2-3, 112*	–	Satpute-Krishnan and Serio (2005)	Figures 1A, 2A,B, 3, 4A,B, 5A
SLL3251	*MATα* [*psi^−^*]*ade1-14 his3Δ200 trp1-289 ura3-52 leu2-3, 112*	–	Satpute-Krishnan and Serio (2005)	Figures 1A, 2A, 3, 5A
SY1220	*MATα* [*PSI^+^*]*^Weak^ ade1-14 his3Δ200 trp1-289 ura3-52 leu2-3, 112*	–	Derdowski et al. (2010)	Figures 1A, 2A, 3, 4A,B, 5A
SLL3260	*MATa/α* [*PSI^+^*]*^Strong^ ade1-14 his3Δ200 trp1–289 ura3-52 leu2-3, 112*	–	This study	
SLL3261	*MATa/α* [*psi^−^*]*ade1-14 his3Δ200 trp1-289 ura3-52 leu2-3, 112*	–	Pei et al. (2017)	
SY3619	*MATa/α* [*PSI^+^*]*^Weak^ ade1-14 his3Δ200 trp1-289 ura3-52 leu2-3, 112*	–	This study	
SY3522	*MATa* [*PSI^+^*]*^Strong^ Δade1-14 his3Δ200 trp1–289 ura3-52 leu2-3, 112*	–	This study	Figure 1A
SY3523	*MATa* [*PSI^+^*]*^Weak^ Δade1-14 his3Δ200 trp1–289 ura3-52 leu2-3, 112*	–	This study	Figure 1A
SY3524	*MATa* [*psi^−^*] *Δade1-14 his3Δ200 trp1–289 ura3-52 leu2-3, 112*	–	This study	Figure 1A
SY597	*MATa* [*PSI^+^*]*^Strong^ ade1–14 trp1–289 his3Δ200::HIS3::P_MFA1_-SUP35-GFP ura3–52 leu2–3,112*	SB117	Satpute-Krishnan et al. (2007)	Figures 6B,C
SY932	*MATa* [*PSI^+^*]*^Weak^ ade1–14 trp1–289 his3Δ200::HIS3::P_MFA1_-SUP35-GFP ura3–52 leu2–3,112*	SB117	This study	Figures 4A, 5A
SY2201	*MATa* [*PSI^+^*]*^Weak^ ade1–14 trp1–289 his3Δ200::HIS3::P_MFA1_-SUP35(G20D)-GFP ura3–52 leu2–3,112*	SB869	This study	Figure 4B and Supplementary Figure S2
SY2203	*MATa* [*PSI^+^*]*^Strong^ ade1–14 trp1–289 his3Δ200::HIS3::P_MFA1_-SUP35(G20D)-GFP ura3–52 leu2–3,112*	SB869	This study	Supplementary Figure S2
SY2393	*MATα* [*psi^−^*] *ade1-14 his3Δ200 trp1–289:: TRP1:: pGPD-GST(UGA)YFPNLS ura3-52 leu2-3, 112*	SB910	Langlois et al. (2016)	Supplementary Figure S1
SY2686	*MATα* [*PSI^+^*]*^Weak^ ade1-14 his3Δ200 trp1–289:: TRP1:: pGPD-GST(UGA)YFPNLS ura3-52 leu2-3, 112*	SB910	This study	Figures 1B,C
SY3088	*MATa* [*psi^−^*] *ade1-14 his3Δ200 trp1-289::TRP1::P_tetO7_-SUP35(G20D)GFP leu2-3,112 ura3–52*	SB1047	This study	Supplementary Figure S2
SY3193	*MATa* [*PSI^+^*]*^Weak^ ade1-14 his3Δ200 trp1289::TRP1::P_tetO7_-SUP35(G20D)GFP leu2-3,112 ura3–52*	SB1047	This study	Figure 2C
SY3421	*MATa* [*PSI^+^*]^Strong^ *ade1–14 trp1–289 his3Δ200::HIS3::P_MFA1_-SUP35(11-61)mCherry ura3–52 leu2–3,112*	SB1120	This study	Supplementary Figure S2
SY3423	*MATa* [*PSI^+^*]*^Weak^ ade1–14 trp1–289 his3Δ200::HIS3::P_MFA1_-SUP35(11-61)mCherry ura3–52 leu2–3,112*	SB1120	This study	Supplementary Figure S2
SY3446	*MATa* [*PSI^+^*]*^Strong^ ade1–14 trp1–289 his3Δ200::HIS3::P_MFA1_-SUP35(11–61)-GFP ura3–52 leu2–3,112*	SB1133	This study	Figure 6C

SY3522, SY3523 and SY3524 were constructed by disrupting *ADE1* in SLL3260, SY3619, and SLL3251, respectively, with a PCR-generated *ADE1* knock-out cassette amplified from pFA6aKanMX4 (Longtine et al., 1998) using ADE13’KO and ADE15’KO. Strains were screened for *ADE1* disruption by PCR of genomic DNA using primers Ade15’Ch and Ade13’Ch, sporulated and verified for 2:2 segregation of the appropriate marker and colony color phenotype. SY3619 was constructed by mating SLL2600 and SY3609; the latter strain was obtained via a mating-type switch of SLL2600. SY3260 was constructed by mating SLL2119 to SY3250. Construction of SY597 has been previously described (Satpute-Krishnan et al., 2007), and the corresponding [*PSI^+^*]^Weak^ strain, SY932 was constructed by integrating *Eco47III*-digested SB117 into SY1220. SY2201 and SY2203 were constructed by integrating *Eco47III*-digested SB869 in SLL2600 and SLL2606, respectively. SY3088 and SY3193 were constructed by integrating a *PpuMI*-digested SB1047 in SLL2119 and SLL2600, respectively. SY2393 and SY2686 were constructed by integrating a *Bsu36I*-digested SB910 in SLL3251 and SY1220, respectively. SY3421 and SY3423 were constructed by integrating *Eco47III*-digested SB1120 into SLL2600 and SLL2606, respectively. SY3446 was constructed by integration of *Eco47III*-digested SB1133 in SY2606. All strains expressing GFP and mCherry were screened for fluorescence by microscopy.

### 4.3 General growth conditions

All strains were grown in YPAD medium [2% dextrose Fisher Scientific, D16), 1% Bactopeptone (Gibco, 211,677), 1% yeast extract (Millipore, 1,037,530,500)] supplemented with 3 mM adenine (Sigma-Aldrich, A8626) unless otherwise specified. All overnight cultures were grown in a shaking incubator at 30°C and maintained at a growth of OD_600_ less than 0.5 for at least 10 doublings to ensure that all experiments were conducted on cells undergoing exponential growth.

### 4.4 Growth conditions for crosses

Liquid cultures of opposite mating types (*MAT***a** and *MAT*α) were grown overnight in YPAD and mated while in exponential growth phase (OD_600_ 0.1–0.2). Cells were harvested, washed once in synthetic complete (SC) medium [0.67% yeast nitrogen base without amino acids (Difco, 291,920), 2% dextrose (Fisher Scientific, D16)] supplemented with auxotrophic amino acids and 2.5 mM adenine (Sigma-Aldrich, A8626) and then mixed on solid synthetic complete medium containing 2.5 mM adenine. To inhibit fragmentation, cells were mixed on extra-rich (i.e., 4% glucose) solid SC medium containing 2.5 mM adenine and 3 mM guanidine hydrochloride (GdnHCl; Sigma-Aldrich, G3273).

### 4.5 Crosses with Δ*ade* strains

Strains were cultured in liquid SC medium containing 15 mg/L adenine (Sigma-Aldrich, A8626) overnight and harvested in early log phase in the morning. Strains of opposite mating type were juxtaposed on SC solid medium for <30 min, mixed to commence mating, then transferred to SC solid medium with reduced adenine (5 mg/L). After ~4 h, patched mated mixtures were transferred to SC solid medium containing 2.5 mg/L adenine and 200 mg/L cytosine (Sigma-Aldrich, C3506) and a pre-embedded microscope slide. Individual zygotes were micromanipulated to the area over the microscope slide. Zygotes were incubated at 30°C in a humidified enclosure for 2 days before the slide was cut out for imaging. Microcolonies were inspected by microscopy, and the number of cells were counted.

### 4.6 Persistence of [*PSI^+^*]^Weak^ aggregates

Zygotes expressing Sup35(G20D)-GFP from P*_tetO7_* were transferred to minimal medium containing 10 μg/mL doxycycline (Sigma-Aldrich, D9891) for 12 h. Zygotes were isolated by micromanipulation, and agar pads with zygotes were cut from plates and transferred to microscope slides for imaging.

### 4.7 Propagon counts

The number of propagons per cell/zygote was determined by an *in vivo* colony-based dilution assay, as previously described (Cox et al., 2003). Propagon variants (i.e., [*PSI^+^*]^Strong^ and [*PSI^+^*]^Weak^) were determined by their respective white and pink colony phenotypes and quantified. To assess effects of GdnHCl on propagon counts and to obtain the titrated [*PSI^+^*]^Strong^ strain, [*PSI^+^*]^Strong^ cultures were grown in liquid YPAD medium supplemented with 3 mM GdnHCl (Sigma-Aldrich).

### 4.8 Stop codon readthrough assay

Cultures were grown in liquid SC medium supplemented 2.5 mM adenine (Sigma-Aldrich, A8626) overnight, harvested and incubated in medium conditioned by cells of the opposite mating type for 1 h. In each cross, one mating partner expressed the GST(UGA)-YFP-NLS reporter from an integrated copy of the SB910 plasmid. Equal OD_600_ units of each mating partner were then mixed and incubated on solid SC medium supplemented with 2.5 mM adenine and allowed to mate for 4 h at 30°C. Cells were then resuspended in liquid SC medium supplemented with 2.5 mM adenine and transferred to microscope slides for imaging. Readthrough activity was determined by measuring the fluorescence in zygotes from each cross versus the signal from a control mating between [*psi^−^*] haploids expressing the reporter. The measured activities were normalized to the average readthrough activity from the [*PSI^+^*]^Weak^ to [*psi^−^*] mating.

### 4.9 Fragmentation assay

A [*PSI^+^*]^Weak^ strain expressing Sup35-GFP from P*_MFA1_* was crossed to a [*psi^−^*], [*PSI^+^*]^Weak^ and [*PSI^+^*]^Strong^ in the presence or absence of 3 mM GdnHCl (Sigma-Aldrich, G3273) on solid SC medium and imaged as previously described (Satpute-Krishnan et al., 2007).

### 4.10 Protein analysis

SDS-PAGE and anti-Sup35 immunoblotting were performed as previously described (Satpute-Krishnan and Serio, 2005). Briefly, 5–6 OD_600_ unit equivalents of cells were harvested while in exponential phase and lysed in buffer [10 mM sodium phosphate buffer, pH 7.5, 0.2% SDS (AmericanBio, AB01920), 1% Triton X-100 (Sigma-Aldrich, T9284), 1 mM phenylmethanesulfonyl fluoride (Sigma-Aldrich, P7626), 0.4 M sodium chloride, 5 μg/mL pepstatin A (ThermoScientific, 78,436)] by vortexing with glass beads at 4°C. Two separate aliquots of each lysate were mixed with 4X loading solution with 20% (wt/v) 2-mercaptoethanol (Sigma-Aldrich, M6250) added just prior to use followed by incubation at either 53°C or 100°C for 10 min. The lysates were fractionated on 4–15% acrylamide gels (Mini PROTEAN TGX gels, BioRad, 4,561,083) and transferred to PVDF membranes (Millipore, IPVH00010) for immunoblotting with rabbit anti-Sup35 primary antibody (Satpute-Krishnan and Serio, 2005) followed by incubation with goat anti-rabbit IgG conjugated to Qdot 655 (Life Technologies, Q11422MP) and imaging on a Typhoon Imager (GE Life Sciences). Band intensities were quantified from an 8-bit image of the immunoblot using LI-COR Image Studio.

### 4.11 Imaging

Imaging was performed in SC medium supplemented with 2.5 mM adenine. Images were obtained on a Zeiss AxioImager M2 equipped with a 100x objective. Fluorescence intensity was analyzed using the Zen software package (Zeiss, Germany). Confocal images were acquired on Leica SP8X confocal microscope equipped with a 63x oil immersion objective and LASX software package.

### 4.12 Imaging and fluorescence loss in photobleaching

Experiments were performed on a Nikon TiE stand equipped with Yokogawa Spinning Disk confocal and FRAP/PA unit for perturbations, using NIS Elements software. Fluorescence Images were acquired with a 60X oil immersion objective, 4×4 bin, 488 nm excitation using 10–15% laser power with 1 and 100% transmission for image acquisitions and bleaching, respectively. Fifteen bleach-image iterations were performed for each zygote with bleaching duration of 2.5 s, laser dwell time of 1,000 us, and image acquisitions every 2 s. Mature daughter buds roughly 1/3–1/2 the size of the entire zygote, were chosen, and bleaching area was kept roughly the same for each zygote; zygotes were individually inspected prior to each FLIP acquisition to ensure septation of bud had not occurred. For each image frame, the average fluorescence pixel intensity was recorded in the mother cell, the bleached bud, a nearby reference cell, and the background. Time-lapse fluorescence intensities in the mother cells were normalized to background and reference levels according to published methods (Bolognesi et al., 2015).

### 4.13 Processing of FLIP measurements and estimating fluorescence loss rates

To extract biological information from the FLIP measurements (Bolognesi et al., 2015), we first adjust the raw intensity measurements for the mother cell [m_raw_(*t*)], using the background b_raw_ and reference cell r_raw_ intensities for each measurement time point *t*, as follows:

 1 *Background Correction:*
Subtract the background intensity from the mother and reference cell measurements,
• Corrected mother cell intensity: m_corrected_(*t*) = m_raw_(*t*) – b_raw_(*t*)
• Corrected reference cell intensity: r_corrected_(*t*) = r_raw_(*t*) – b_raw_(*t*)
 2 *Photobleaching Correction:*
Adjust for photobleaching by dividing the corrected mother measurements by the corrected reference intensity:
• Adjusted mother cell intensity:m_adjusted_(*t*) = m_corrected_(*t*)/r_corrected_(*t*)
 3 *Normalization:*
Normalize the adjusted fluorescence intensity in the mother cell to show the fluorescence loss relative to the initial fluorescence measurement. This is done by dividing each adjusted fluorescence measurement made at time *t_i_*, by the initial fluorescence measurement made at time *t*_0_:
• Normalized intensity: M(*t_i_*) = m_adjusted_(*t_i_*)/ m_adjusted_(*t*_0_).

After processing the FLIP measurements for each variant cross, we model the fluorescence loss in the mother relative to the initial fluorescence, M(*t*), as an exponential decay process. The fluorescence loss rate, denoted as λ (with units of per second), is estimated by fitting an exponential decay model exp.( −λ *t*) to each replicate in each cross. This approach allows us to compare fluorescence loss rates between different variant crosses.

### 4.14 Statistical analyses of variant crosses

#### 4.14.1 Fluorescence loss in photobleaching

For consistency and due to differences in the variance of the fluorescence loss estimates, we apply the unequal variance *t*-test when comparing the fluorescence loss between two conditions. This method is used even when there is no statistical evidence of differences in variance between the two groups (Ruxton, 2006). A *p*-value of less than 0.05 is significant statistical evidence of differences in the fluorescence loss between the two crosses being compared.

#### 4.14.2 Propagon counts

When comparing three or more groups of propagon counts, we first perform an analysis of variance (ANOVA). Due to heteroskedasticity between variant crosses and deviations from normality, we utilize the more robust Welch’s ANOVA test to determine if there are differences in mean propagon counts among the crossing being compared (Lantz, 2013). If the ANOVA indicates significant differences, we then conduct pairwise unequal variance *t*-tests for all variant crosses. To reduce type 1 errors (false positives) when comparing more than two groups, we indicate significance with an asterisk for those comparisons that remain significant after applying a Bonferroni correction to adjust our significance level of 0.05 (Nahler, 2009), while reporting all *p*-values.

All computations, calibrations, and statistical analyses were performed using MATLAB R2023a (MathWorks, Inc., Natick, MA, USA). Specifically, for model calibration we used the nlinfit function, and for the unequal variance *t*-test we used the ttest2 function with the ‘vartype’ option set to ‘unequal’.

## Data availability statement

The raw data supporting the conclusions of this article will be made available by the authors, without undue reservation.

## Author contributions

JN: Conceptualization, Formal analysis, Investigation, Methodology, Writing – original draft, Writing – review & editing. NS: Conceptualization, Formal analysis, Investigation, Methodology, Writing – original draft, Writing – review & editing. FS: Conceptualization, Formal analysis, Investigation, Methodology, Writing – review & editing. SS: Conceptualization, Funding acquisition, Project administration, Writing – review & editing. TS: Conceptualization, Funding acquisition, Project administration, Writing – original draft, Writing – review & editing.

## References

[ref1] Allsop D. Ikeda S. Bruce M. Glenner G. G. (1988). Cerebrovascular amyloid in scrapie-affected sheep reacts with antibodies to prion protein. Neurosci. Lett. 92, 234–239. doi: 10.1016/0304-3940(88)90067-5 3185993

[ref2] Astor M. T. Kamiya E. Sporn Z. A. Berger S. E. Hines J. K. (2018). Variant-specific and reciprocal Hsp40 functions in Hsp104-mediated prion elimination. Mol. Microbiol. 109, 41–62. doi: 10.1111/mmi.13966, PMID: 29633387 PMC6099457

[ref3] Atarashi R. Moore R. A. Sim V. L. Hughson A. G. Dorward D. W. Onwubiko H. A.. (2007). Ultrasensitive detection of scrapie prion protein using seeded conversion of recombinant prion protein. Nat. Methods 4, 645–650. doi: 10.1038/nmeth1066, PMID: 17643109

[ref4] Atarashi R. Sim V. L. Nishida N. Caughey B. Katamine S. (2006). Prion strain-dependent differences in conversion of mutant prion proteins in cell culture. J. Virol. 80, 7854–7862. doi: 10.1128/jvi.00424-06 16873242 PMC1563786

[ref5] Bartz J. C. Bessen R. A. McKenzie D. Marsh R. F. Aiken J. M. (2000). Adaptation and selection of prion protein strain conformations following interspecies transmission of transmissible mink encephalopathy. J. Virol. 74, 5542–5547. doi: 10.1128/JVI.74.12.5542-5547.2000, PMID: 10823860 PMC112040

[ref6] Bartz J. C. Kramer M. L. Sheehan M. H. Hutter J. A. Ayers J. I. Bessen R. A.. (2007). Prion interference is due to a reduction in strain-specific PrPSc levels. J. Virol. 81, 689–697. doi: 10.1128/JVI.01751-06, PMID: 17079313 PMC1797475

[ref7] Bartz J. C. Marsh R. F. McKenzie D. I. Aiken J. M. (1998). The host range of chronic wasting disease is altered on passage in ferrets. Virology 251, 297–301. doi: 10.1006/viro.1998.9427, PMID: 9837794

[ref8] Bateman D. A. Wickner R. B. (2013). The [*PSI^+^*] prion exists as a dynamic cloud of variants. PLoS Genet. 9:e1003257. doi: 10.1371/journal.pgen.1003257, PMID: 23382698 PMC3561065

[ref9] Bessen R. A. Marsh R. F. (1992). Biochemical and physical properties of the prion protein from two strains of the transmissible mink encephalopathy agent. J. Virol. 66, 2096–2101. doi: 10.1128/jvi.66.4.2096-2101.1992, PMID: 1347795 PMC289000

[ref1002] Bessen R. A. Marsh R. F. (1994). Distinct PrP properties suggest the molecular basis of strain variation. J. Virol. 68, 7859–7868. doi: 10.1128/jvi.68.12.7859-7868.1994, PMID: 7966576 PMC237248

[ref10] Bolognesi A. Sliwa-Gonzalez A. Prasad R. Barral Y. (2015). Yeast cytokinesis, methods and protocols. Methods Mol. Biol. 1369, 25–44. doi: 10.1007/978-1-4939-3145-3_3 26519303

[ref11] Borchelt D. R. Scott M. Taraboulos A. Stahl N. Prusiner S. B. (1990). Scrapie and cellular prion proteins differ in their kinetics of synthesis and topology in cultured cells. J. Cell Biol. 110, 743–752. doi: 10.1083/jcb.110.3.743, PMID: 1968466 PMC2116048

[ref12] Bradley M. E. Edskes H. K. Hong J. Y. Wickner R. B. Liebman S. W. (2002). Interactions among prions and prion “strains” in yeast. Proc. Natl. Acad. Sci. USA 99, 16392–16399. doi: 10.1073/pnas.152330699, PMID: 12149514 PMC139899

[ref13] Bruce M. E. Dickinson A. G. (1987). Biological evidence that scrapie agent has an independent genome. J. Gen. Virol. 68, 79–89. doi: 10.1099/0022-1317-68-1-79 3100717

[ref14] Burke C. M. Walsh D. J. Mark K. M. K. Deleault N. R. Nishina K. A. Agrimi U.. (2020). Cofactor and glycosylation preferences for in vitro prion conversion are predominantly determined by strain conformation. PLoS Pathog. 16:e1008495. doi: 10.1371/journal.ppat.1008495, PMID: 32294141 PMC7185723

[ref15] Cali I. Castellani R. Alshekhlee A. Cohen Y. Blevins J. Yuan J.. (2009). Co-existence of scrapie prion protein types 1 and 2 in sporadic Creutzfeldt-Jakob disease: its effect on the phenotype and prion-type characteristics. Brain 132, 2643–2658. doi: 10.1093/brain/awp196, PMID: 19734292 PMC2766234

[ref16] Cassard H. Huor A. Espinosa J.-C. Douet J.-Y. Lugan S. Aron N.. (2020). Prions from sporadic Creutzfeldt-Jakob disease patients propagate as strain mixtures. MBio 11, e00393–e00320. doi: 10.1128/mbio.00393-20, PMID: 32546613 PMC7298703

[ref17] Caughey B. Raymond G. J. (1991). The scrapie-associated form of PrP is made from a cell surface precursor that is both protease- and phospholipase-sensitive. J. Biol. Chem. 266, 18217–18223. doi: 10.1016/S0021-9258(18)55257-1, PMID: 1680859

[ref18] Chernoff Y. O. Lindquist S. L. Ono B. Inge-Vechtomov S. G. Liebman S. W. (1995). Role of the chaperone protein Hsp104 in propagation of the yeast prion-like factor [*PSI^+^*]. Science 268, 880–884. doi: 10.1126/science.7754373, PMID: 7754373

[ref19] Chiti F. Dobson C. M. (2006). Protein misfolding, functional amyloid, and human disease. Annu. Rev. Biochem. 75, 333–366. doi: 10.1146/annurev.biochem.75.101304.123901 16756495

[ref20] Chiti F. Dobson C. M. (2016). Protein Misfolding, amyloid formation, and human disease: a summary of Progress over the last decade. Annu. Rev. Biochem. 86, 27–68. doi: 10.1146/annurev-biochem-061516-045115 28498720

[ref21] Chiti F. Stefani M. Taddei N. Ramponi G. Dobson C. M. (2003). Rationalization of the effects of mutations on peptide andprotein aggregation rates. Nature 424, 805–808. doi: 10.1038/nature01891, PMID: 12917692

[ref22] Collinge J. (2001). Prion diseases of humans and animals: their causes and molecular basis. Annu. Rev. Neurosci. 24, 519–550. doi: 10.1146/annurev.neuro.24.1.519 11283320

[ref23] Collinge J. Clarke A. R. (2007). A general model of prion strains and their pathogenicity. Science 318, 930–936. doi: 10.1126/science.1138718 17991853

[ref24] Cox B. Ness F. Tuite M. (2003). Analysis of the generation and segregation of propagons: entities that propagate the [*PSI^+^*] prion in yeast. Genetics 165, 23–33. doi: 10.1093/genetics/165.1.23, PMID: 14504215 PMC1462756

[ref25] Derdowski A. Sindi S. S. Klaips C. L. DiSalvo S. Serio T. R. (2010). A size threshold limits prion transmission and establishes phenotypic diversity. Science 330, 680–683. doi: 10.1126/science.1197785, PMID: 21030659 PMC3003433

[ref26] Derkatch I. L. Bradley M. E. Zhou P. Liebman S. W. (1999). The PNM2 mutation in the prion protein domain of *SUP35* has distinct effects on different variants of the [*PSI^+^*] prion in yeast. Curr. Genet. 35, 59–67. doi: 10.1007/s002940050433, PMID: 10079323

[ref27] Derkatch I. L. Chernoff Y. O. Kushnirov V. V. Inge-Vechtomov S. G. Liebman S. W. (1996). Genesis and variability of [*PSI*] prion factors in *Saccharomyces cerevisiae*. Genetics 144, 1375–1386. doi: 10.1093/genetics/144.4.1375, PMID: 8978027 PMC1207691

[ref28] Diaz-Avalos R. King C.-Y. Wall J. Simon M. Caspar D. L. D. (2005). Strain-specific morphologies of yeast prion amyloid fibrils. P Natl. Acad. Sci. U. S. A. 102, 10165–10170. doi: 10.1073/pnas.0504599102, PMID: 16006506 PMC1177419

[ref29] Diaz-Espinoza R. Morales R. Concha-Marambio L. Moreno-Gonzalez I. Moda F. Soto C. (2018). Treatment with a non-toxic, self-replicating anti-prion delays or prevents prion disease in vivo. Mol. Psychiatry 23, 777–788. doi: 10.1038/mp.2017.84, PMID: 28630454 PMC5738294

[ref30] Dickinson A. G. Fraser H. Meikle V. M. Outram G. W. (1972). Competition between different scrapie agents in mice. Nat. New Biol. 237, 244–245. doi: 10.1038/newbio237244a0, PMID: 4624846

[ref31] Dickinson A. Outram G. (1979). “The scrapie replication-site hypothesis and its implications for pathogenesis” in Slow transmissible diseases of the central nervous system. eds. Prusiner S. Hadlow W. (New York: Academic), 13–31.

[ref32] DiSalvo S. Derdowski A. Pezza J. A. Serio T. R. (2011). Dominant prion mutants induce curing through pathways that promote chaperone-mediated disaggregation. Nat. Struct. Mol. Biol. 18, 486–492. doi: 10.1038/nsmb.2031 21423195 PMC3082495

[ref33] Doel S. M. McCready S. J. Nierras C. R. Cox B. S. (1994). The dominant PNM2- mutation which eliminates the psi factor of *Saccharomyces cerevisiae* is the result of a missense mutation in the *SUP35* gene. Genetics 137, 659–670. doi: 10.1093/genetics/137.3.659, PMID: 8088511 PMC1206025

[ref34] Eckland T. E. Shikiya R. A. Bartz J. C. (2018). Independent amplification of co-infected long incubation period low conversion efficiency prion strains. PLoS Pathog. 14:e1007323. doi: 10.1371/journal.ppat.1007323, PMID: 30335854 PMC6193734

[ref35] Ferreira P. C. Ness F. Edwards S. R. Cox B. S. Tuite M. F. (2001). The elimination of the yeast [*PSI^+^*] prion by guanidine hydrochloride is the result of Hsp104 inactivation. Mol. Microbiol. 40, 1357–1369. doi: 10.1046/j.1365-2958.2001.02478.x 11442834

[ref36] Fitzpatrick D. A. O’Brien J. Moran C. Hasin N. Kenny E. Cormican P.. (2011). Assessment of inactivating stop codon mutations in forty *Saccharomyces cerevisiae* strains: implications for [*PSI^+^*] prion-mediated phenotypes. PLoS One 6:e28684. doi: 10.1371/journal.pone.0028684, PMID: 22194885 PMC3240633

[ref37] Galgoczy D. J. Cassidy-Stone A. Llinas M. O’Rourke S. M. Herskowitz I. DeRisi J. L.. (2004). Genomic dissection of the cell-type-specification circuit in *Saccharomyces cerevisiae*. Proc. Natl. Acad. Sci. U. S. A. 101, 18069–18074. doi: 10.1073/pnas.0407611102, PMID: 15604142 PMC535907

[ref38] Gao X. Carroni M. Nussbaum-Krammer C. Mogk A. Nillegoda N. B. Szlachcic A.. (2015). Human Hsp70 Disaggregase reverses Parkinson’s-linked α-Synuclein amyloid fibrils. Mol. Cell 59, 781–793. doi: 10.1016/j.molcel.2015.07.012, PMID: 26300264 PMC5072489

[ref39] Ghaemmaghami S. Ahn M. Lessard P. Giles K. Legname G. DeArmond S. J.. (2009). Continuous quinacrine treatment results in the formation of drug-resistant prions. PLoS Pathog. 5:e1000673. doi: 10.1371/journal.ppat.1000673, PMID: 19956709 PMC2777304

[ref40] Grimminger V. Richter K. Imhof A. Buchner J. Walter S. (2004). The prion curing agent guanidinium chloride specifically inhibits ATP hydrolysis by Hsp104. J. Biol. Chem. 279, 7378–7383. doi: 10.1074/jbc.M312403200 14668331

[ref41] Higurashi T. Hines J. K. Sahi C. Aron R. Craig E. A. (2008). Specificity of the J-protein Sis1 in the propagation of 3 yeast prions. Proc. Natl. Acad. Sci. U. S. A. 105, 16596–16601. doi: 10.1073/pnas.0808934105, PMID: 18955697 PMC2575465

[ref42] Hope J. Reekie L. J. D. Hunter N. Multhaup G. Beyreuther K. White H.. (1988). Fibrils from brains of cows with new cattle disease contain scrapie-associated protein. Nature 336, 390–392. doi: 10.1038/336390a0, PMID: 2904126

[ref43] Jucker M. Walker L. C. (2013). Self-propagation of pathogenic protein aggregates in neurodegenerative diseases. Nature 501, 45–51. doi: 10.1038/nature12481, PMID: 24005412 PMC3963807

[ref44] Jung G. Masison D. C. (2001). Guanidine hydrochloride inhibits Hsp104 activity in vivo: a possible explanation for its effect in curing yeast prions. Curr. Microbiol. 43, 7–10. doi: 10.1007/s002840010251, PMID: 11375656

[ref45] Kimberlin R. H. Walker C. A. (1978). Evidence that the transmission of one source of scrapie agent to hamsters involves separation of agent strains from a mixture. J. Gen. Virol. 39, 487–496. doi: 10.1099/0022-1317-39-3-487 96212

[ref46] Kimberlin R. H. Walker C. A. (1985). Competition between strains of scrapie depends on the blocking agent being infectious. Intervirology 23, 74–81. doi: 10.1159/000149588 3920169

[ref47] King C.-Y. (2001). Supporting the structural basis of prion strains: induction and identification of [PSI] variants. J. Mol. Biol. 307, 1247–1260. doi: 10.1006/jmbi.2001.4542, PMID: 11292339

[ref48] Kitamoto T. Tateishi J. Tashima T. Takeshita I. Barry R. A. Dearmond S. J.. (1986). Amyloid plaques in Creutzfeldt-Jakob disease stain with prion protein antibodies. Ann. Neurol. 20, 204–208. doi: 10.1002/ana.410200205 3092727

[ref49] Knowles T. P. J. Vendruscolo M. Dobson C. M. (2014). The amyloid state and its association with protein misfolding diseases. Nat. Rev. Mol. Cell Biol. 15, 384–396. doi: 10.1038/nrm3810 24854788

[ref50] Langlois C. R. Pei F. Sindi S. S. Serio T. R. (2016). Distinct prion domain sequences ensure efficient amyloid propagation by promoting chaperone binding or processing in vivo. PLoS Genet. 12:e1006417. doi: 10.1371/journal.pgen.1006417, PMID: 27814358 PMC5096688

[ref51] Lantz B. (2013). The impact of sample non-normality on ANOVA and alternative methods. Br. J. Math. Stat. Psychol. 66, 224–244. doi: 10.1111/j.2044-8317.2012.02047.x, PMID: 22624658

[ref52] Li J. Browning S. Mahal S. P. Oelschlegel A. M. Weissmann C. (2010). Darwinian evolution of prions in cell culture. Science 327, 869–872. doi: 10.1126/science.1183218, PMID: 20044542 PMC2848070

[ref53] Lin J.-Y. Liao T.-Y. Lee H.-C. King C.-Y. (2011). Inter-allelic prion propagation reveals conformational relationships among a multitude of [*PSI*] strains. PLoS Genet. 7:e1002297. doi: 10.1371/journal.pgen.1002297, PMID: 21980301 PMC3183073

[ref54] Longtine M. S. McKenzie A. 3rd DeMarini D. J. Shah N. G. Wach A. Brachat A.. (1998). Additional modules for versatile and economical PCR-based gene deletion and modification in *Saccharomyces cerevisiae*. Yeast 14, 953–961. doi: 10.1002/(SICI)1097-0061(199807)14:10<953::AID-YEA293>3.0.CO;2-U, PMID: 9717241

[ref1003] Makarava N. Ostapchenko V. G. Savtchenko R. Baskakov I. V. (2009). Conformational switching within individual amyloid fibers. J. Biol. Chem. 284, 14386–14395. doi: 10.1074/jbc.M900533200, PMID: 19329794 PMC2682887

[ref55] Manuelidis L. (1998). Vaccination with an attenuated Creutzfeldt-Jakob disease strain prevents expression of a virulent agent. Proc. Natl. Acad. Sci. U. S. A. 95, 2520–2525. doi: 10.1073/pnas.95.5.2520 9482918 PMC19398

[ref56] Manuelidis L. Lu Z. Y. (2003). Virus-like interference in the latency and prevention of Creutzfeldt-Jakob disease. Proc. Natl. Acad. Sci. 100, 5360–5365. doi: 10.1073/pnas.0931192100, PMID: 12692308 PMC154350

[ref57] Masel J. Jansen V. A. Nowak M. A. (1999). Quantifying the kinetic parameters of prion replication. Biophys. Chem. 77, 139–152. doi: 10.1016/S0301-4622(99)00016-2, PMID: 10326247

[ref58] McKinley M. P. Bolton D. C. Prusiner S. B. (1983). A protease-resistant protein is a structural component of the scrapie prion. Cell 35, 57–62. doi: 10.1016/0092-8674(83)90207-6, PMID: 6414721

[ref59] Naeimi W. R. Serio T. R. (2022). Beyond amyloid fibers: accumulation, biological relevance, and regulation of higher-order prion architectures. Viruses 14:1635. doi: 10.3390/v14081635 35893700 PMC9332770

[ref60] Nahler G. (2009). Bonferroni correction. Dictionary Pharmaceut. Med. 18. doi: 10.1007/978-3-211-89836-9_140

[ref61] Nelson R. Sawaya M. R. Balbirnie M. Madsen A. O. Riekel C. Grothe R.. (2005). Structure of the cross-beta spine of amyloid-like fibrils. Nature 435, 773–778. doi: 10.1038/nature03680, PMID: 15944695 PMC1479801

[ref62] Nowak M. A. Krakauer D. C. Klug A. May R. M. (1998). Prion infection dynamics. Integrat. Biol. 1, 3–15. doi: 10.1002/(sici)1520-6602(1998)1:1<3::aid-inbi2>3.0.co;2-9

[ref63] Oelschlegel A. M. Weissmann C. (2013). Acquisition of drug resistance and dependence by prions. PLoS Pathog. 9:e1003158. doi: 10.1371/journal.ppat.1003158, PMID: 23408888 PMC3567182

[ref64] Pan K. M. Baldwin M. A. Nguyen J. Gasset M. Serban A. Grothe D.. (1993). Conversion of alpha-helices into beta-sheet features in the formation of the scrapie prion proteins. Proc. Natl. Acad. Sci. USA 90, 10962–10966. doi: 10.1073/pnas.90.23.10962 7902575 PMC47901

[ref65] Parchi P. Strammiello R. Notari S. Giese A. Langeveld J. P. Ladogana A.. (2009). Incidence and spectrum of sporadic Creutzfeldt-Jakob disease variants with mixed phenotype and co-occurrence of PrPSc types: an updated classification. Acta Neuropathol. 118, 659–671. doi: 10.1007/s00401-009-0585-1, PMID: 19718500 PMC2773124

[ref66] Pei F. DiSalvo S. Sindi S. S. Serio T. R. (2017). A dominant-negative mutant inhibits multiple prion variants through a common mechanism. PLoS Genet. 13:e1007085. doi: 10.1371/journal.pgen.1007085, PMID: 29084237 PMC5679637

[ref67] Pezza J. A. Villali J. Serio T. R. (2014). Amyloid-associated activity contributes to the severity and toxicity of a prion phenotype. Nat. Commun. 5:4384. doi: 10.1038/ncomms5384 25023996 PMC4156856

[ref68] Polymenidou M. Stoeck K. Glatzel M. Vey M. Bellon A. Aguzzi A. (2005). Coexistence of multiple PrPSc types in individuals with Creutzfeldt-Jakob disease. Lancet Neurol. 4, 805–814. doi: 10.1016/s1474-4422(05)70225-8, PMID: 16297838

[ref69] Roberts B. E. Duennwald M. L. Wang H. Chung C. Lopreiato N. P. Sweeny E. A.. (2009). A synergistic small-molecule combination directly eradicates diverse prion strain structures. Nat. Chem. Biol. 5, 936–946. doi: 10.1038/nchembio.246, PMID: 19915541 PMC2909773

[ref70] Ruxton G. (2006). The unequal variance t-test is an underused alternative to Student's t-test and the Mann–Whitney U test. Behav. Ecol. 17, 688–690. doi: 10.1093/beheco/ark016

[ref71] Sadlish H. Rampelt H. Shorter J. Wegrzyn R. D. Andréasson C. Lindquist S.. (2008). Hsp110 chaperones regulate prion formation and propagation in *S. cerevisiae by two discrete activities*. PLoS One 3:e1763. doi: 10.1371/journal.pone.0001763, PMID: 18335038 PMC2258148

[ref72] Santoso A. Chien P. Osherovich L. Z. Weissman J. S. (2000). Molecular basis of a yeast prion species barrier. Cell 100, 277–288. doi: 10.1016/s0092-8674(00)81565-2, PMID: 10660050

[ref73] Satpute-Krishnan P. Langseth S. X. Serio T. R. (2007). Hsp104-dependent remodeling of prion complexes mediates protein-only inheritance. PLoS Biol. 5:e24. doi: 10.1371/journal.pbio.0050024, PMID: 17253904 PMC1779812

[ref74] Satpute-Krishnan P. Serio T. R. (2005). Prion protein remodelling confers an immediate phenotypic switch. Nature 437, 262–265. doi: 10.1038/nature03981, PMID: 16148935

[ref75] Schutt C. R. Bartz J. C. (2008). Prion interference with multiple prion isolates. Prion 2, 61–63. doi: 10.4161/pri.2.2.6806, PMID: 19098442 PMC2634519

[ref76] Serio T. R. Lindquist S. L. (1999). [*PSI^+^*]: an epigenetic modulator of translation termination efficiency. Cell Dev. Biol. 15, 661–703. doi: 10.1146/annurev.cellbio.15.1.661, PMID: 10611975

[ref77] Sharma J. Liebman S. W. (2012). [*PSI^+^*] prion variant establishment in yeast. Mol. Microbiol. 86, 866–881. doi: 10.1111/mmi.12024, PMID: 22998111 PMC3543502

[ref78] Shikiya R. A. Ayers J. I. Schutt C. R. Kincaid A. E. Bartz J. C. (2010). Coinfecting prion strains compete for a limiting cellular resource. J. Virol. 84, 5706–5714. doi: 10.1128/jvi.00243-10 20237082 PMC2876617

[ref79] Shikiya R. A. Bartz J. C. (2023). “Prion strain interference” in Prions and diseases. eds. Zou W.-Q. Gambetti P. (Springer Cham), 107–122.

[ref80] Shorter J. (2010). Emergence and natural selection of drug-resistant prions. Mol. BioSyst. 6, 1115–1130. doi: 10.1039/c004550k, PMID: 20422111 PMC2936920

[ref81] Silveira J. R. Raymond G. J. Hughson A. G. Race R. E. Sim V. L. Hayes S. F.. (2005). The most infectious prion protein particles. Nature 437, 257–261. doi: 10.1038/nature03989, PMID: 16148934 PMC1513539

[ref82] Song Y. Wu Y.-X. Jung G. Tutar Y. Eisenberg E. Greene L. E.. (2005). Role for Hsp70 chaperone in *Saccharomyces cerevisiae* prion seed replication. Eukaryot. Cell 4, 289–297. doi: 10.1128/ec.4.2.289-297.2005, PMID: 15701791 PMC549339

[ref83] Tanaka M. Collins S. R. Toyama B. H. Weissman J. S. (2006). The physical basis of how prion conformations determine strain phenotypes. Nature 442, 585–589. doi: 10.1038/nature04922, PMID: 16810177

[ref1004] Terry C. Wenborn A. Gros N. Sells J. Joiner S. Hosszu L. L.. (2016). Ex vivo mammalian prions are formed of paired double helical prion protein fibrils. Open Biol. 6:160035. doi: 10.1098/sob.160035, PMID: 27249641 PMC4892434

[ref84] Tipton K. A. Verges K. J. Weissman J. S. (2008). *In vivo* monitoring of the prion replication cycle reveals a critical role for Sis1 in delivering substrates to Hsp104. Mol. Cell 32, 584–591. doi: 10.1016/j.molcel.2008.11.003, PMID: 19026788 PMC2875781

[ref85] Toyama B. H. Kelly M. J. S. Gross J. D. Weissman J. S. (2007). The structural basis of yeast prion strain variants. Nature 449, 233–237. doi: 10.1038/nature06108, PMID: 17767153

[ref86] Tuite M. F. Serio T. R. (2010). The prion hypothesis: from biological anomaly to basic regulatory mechanism. Nat. Rev. Mol. Cell Biol. 11, 823–833. doi: 10.1038/nrm3007, PMID: 21081963 PMC3003427

[ref87] Villali J. Dark J. Brechtel T. M. Pei F. Structural S. S. N. (2020). (2020). Nucleation seed size determines amyloid clearance and establishes a barrier to prion appearance in yeast. Nat. Struct. Mol. Biol. 27, 540–549. doi: 10.1038/s41594-020-0416-6, PMID: 32367069 PMC7293557

[ref88] Walker L. C. Diamond M. I. Duff K. E. Hyman B. T. (2013). Mechanisms of protein seeding in neurodegenerative diseases. JAMA Neurol. 70, 304–310. doi: 10.1001/jamaneurol.2013.1453, PMID: 23599928 PMC3665718

[ref89] Wickner R. B. (2016). Yeast and fungal prions. Cold Spring Harb. Perspect. Biol. 8:a023531. doi: 10.1101/cshperspect.a023531 27481532 PMC5008071

[ref90] Wickner R. B. Dyda F. Tycko R. (2008). Amyloid of Rnq1p, the basis of the [*PIN^+^*] prion, has a parallel in-register β-sheet structure. Proc. Natl. Acad. Sci. 105, 2403–2408. doi: 10.1073/pnas.0712032105, PMID: 18268327 PMC2268149

[ref91] Yu C.-I. King C.-Y. (2018). Forms and abundance of chaperone proteins influence yeast prion variant competition. Mol. Microbiol. 111, 798–810. doi: 10.1111/mmi.14192, PMID: 30582872

