# Supplementary material for: Multiple aspects of amyloid dynamics in vivo integrate to establish prion variant dominance in yeast
Source: Front Mol Neurosci. 2024 Jul 30;17:1439442. doi: 10.3389/fnmol.2024.1439442 (PMC11319303; doi:10.3389/fnmol.2024.1439442)
Supplement: Supplementary file 5 [file Table1.DOCX]

**Supplementary Table S1: p-values for Budded Cells from Crosses in Figure 1A**

|  | Δ*ade1* [*psi^-^*] X [*psi^-^*] | Δ*ade1* [*psi^-^*] X [*PSI^+^*]^Weak^ | Δ*ade1* [*psi^-^*] X [*PSI^+^*]^Strong^ | Δ*ade1* [*PSI^+^*]^Weak^ X [*psi^-^*] | Δ*ade1* [*PSI^+^*]^Weak^ X [*PSI^+^*]^Weak^ | Δ*ade1* [*PSI^+^*]^Weak^ X [*PSI^+^*]^Strong^ | Δ*ade1* [*PSI^+^*]^Strong^ X [*psi^-^*] | Δ*ade1* [*PSI^+^*]^Strong^ X [*PSI^+^*]^Weak^ | Δ*ade1* [*PSI^+^*]^Strong^ X [*PSI^+^*]^Strong^ |
| --- | --- | --- | --- | --- | --- | --- | --- | --- | --- |
| Δ*ade1*[*psi^-^*] X [*psi^-^*] |  | 1.50E-03* | 1.29E-06* | 6.576E-01 | 9.199E-01 | 9.93E-07* | 3.60E-11* | 3.53E-10* | 1.57E-12* |
| Δ*ade1*[*psi^-^*] X [*PSI^+^*]^Weak^ |  |  | 8.11E-05* | 4.15E-05* | 8.43E-04* | 3.51E-04* | 3.03E-07* | 7.30E-06* | 3.04E-08* |
| Δ*ade1*[*psi^-^*] X [*PSI^+^*]^Strong^ |  |  |  | 7.45E-07* | 1.11E-06* | 2.351E-01 | 2.776E-01 | 7.456E-02 | 3.011E-01 |
| Δ*ade1*[*PSI^+^*]^Weak^ X [*psi^-^*] |  |  |  |  | 7.377E-01 | 3.29E-07* | 4.99E-12* | 1.89E-11* | 1.18E-13* |
| Δ*ade1*[*PSI^+^*]^Weak^ X [*PSI^+^*]^Weak^ |  |  |  |  |  | 7.64E-07* | 1.82E-11* | 1.51E-10* | 6.07E-13* |
| Δ*ade1*[*PSI^+^*]^Weak^ X [*PSI^+^*]^Strong^ |  |  |  |  |  |  | 7.633E-01 | 5.783E-01 | 6.911E-01 |
| Δ*ade1*[*PSI^+^*]^Strong^ X [*psi^-^*] |  |  |  |  |  |  |  | 2.580E-01 | 9.084E-01 |
| Δ*ade1*[*PSI^+^*]^Strong^ X [*PSI^+^*]^Weak^ |  |  |  |  |  |  |  |  | 1.905E-01 |
| Δ*ade1*[*PSI^+^*]^Strong^ X [*PSI^+^*]^Strong^ |  |  |  |  |  |  |  |  |  |

*indicates statistical significance
