# Supplementary material for: Multiple aspects of amyloid dynamics in vivo integrate to establish prion variant dominance in yeast
Source: Front Mol Neurosci. 2024 Jul 30;17:1439442. doi: 10.3389/fnmol.2024.1439442 (PMC11319303; doi:10.3389/fnmol.2024.1439442)
Supplement: Supplementary file 6 [file Table2.DOCX]

**Supplementary Table S2: p-values for Fluorescence Intensity from Crosses in Figure 1C**

|  | [*psi^-^*] X [*PSI^+^*]^Weak^ | [*PSI^+^*]^Weak^ X [*PSI^+^*]^Weak^ | [*PSI^+^*]^Strong^ X [*PSI^+^*]^Weak^ |
| --- | --- | --- | --- |
| [*psi^-^*] X [*PSI^+^*]^Weak^ |  | 1.20E-02* | 1.20E-05* |
| [*PSI^+^*]^Weak^ X [*PSI^+^*]^Weak^ |  |  | 2.439E-02 |
| [*PSI^+^*]^Strong^ X [*PSI^+^*]^Weak^ |  |  |  |

*indicates statistical significance
