# Supplementary material for: Multiple aspects of amyloid dynamics in vivo integrate to establish prion variant dominance in yeast
Source: Front Mol Neurosci. 2024 Jul 30;17:1439442. doi: 10.3389/fnmol.2024.1439442 (PMC11319303; doi:10.3389/fnmol.2024.1439442)
Supplement: Supplementary file 7 [file Table3.DOCX]

**Supplementary Table S3: p-values for [*PSI^+^*]^Strong^** **Propagons from Crosses in Figure 2A**

|  | [*PSI^+^*]^Strong^  Haploid | [*PSI^+^*]^Strong^ X [*psi^-^*] | [*PSI^+^*]^Strong^ x [*psi^-^*]  (+ GdnHCl) | [*PSI^+^*]^Strong^ titrated X [*psi^-^*] | [*PSI^+^*]^Strong^ X [*PSI^+^*]^Strong^ | [*PSI^+^*]^Strong^ X [*PSI^+^*]^Strong^  (+ GdnHCl) | [*PSI^+^*]^Strong^ titrated X [*PSI^+^*]^Strong^ | [*PSI^+^*]^Strong^ X [*PSI^+^*]^Weak^ | [*PSI^+^*]^Strong^ X [*PSI^+^*]^Weak^  (+ GdnHCl) | [*PSI^+^*]^Strong^ titrated X [*PSI^+^*]^Weak^ |
| --- | --- | --- | --- | --- | --- | --- | --- | --- | --- | --- |
| [*PSI^+^*]^Strong^  Haploid |  | 1.24E-07* | 2.75E-04* | 1.62E-05* | 3.30E-05* | 3.433E-02 | 5.27E-06* | 2.55E-06* | 1.03E-05* | 5.282E-02 |
| [*PSI^+^*]^Strong^ X [*psi^-^*] |  |  | 6.58E-09* | 2.112E-01 | 1.361E-01 | 6.71E-09* | 3.375E-01 | 2.553E-01 | 2.53E-09* | 5.54E-09* |
| [*PSI^+^*]^Strong^ X [*psi^-^*]  (+ GdnHCl) |  |  |  | 8.56E-07* | 2.13E-06* | 3.650E-01 | 2.88E-07* | 1.95E-07* | 1.993E-01 | 5.224E-01 |
| [*PSI^+^*]^Strong^ titrated X [*psi^-^*] |  |  |  |  | 8.312E-01 | 1.40E-06* | 7.591E-01 | 7.799E-01 | 3.39E-07* | 1.21E-06* |
| [*PSI^+^*]^Strong^ X [*PSI^+^*]^Strong^ |  |  |  |  |  | 2.95E-06* | 5.966E-01 | 5.976E-01 | 9.02E-07* | 2.38E-06* |
| [*PSI^+^*]^Strong^ X [*PSI^+^*]^Strong^  (+ GdnHCl) |  |  |  |  |  |  | 4.12E-07* | 1.21E-07* | 6.511E-02 | 9.019E-01 |
| [*PSI^+^*]^Strong^ titrated X [*PSI^+^*]^Strong^ |  |  |  |  |  |  |  | 9.503E-01 | 1.14E-07* | 3.44E-07* |
| [*PSI^+^*]^Strong^ X [*PSI^+^*]^Weak^ |  |  |  |  |  |  |  |  | 8.41E-08* | 8.60E-08* |
| [*PSI^+^*]^Strong^ X [*PSI^+^*]^Weak^  (+ GdnHCl) |  |  |  |  |  |  |  |  |  | 1.420E-01 |
| [*PSI^+^*]^Strong^ titrated X [*PSI^+^*]^Weak^ |  |  |  |  |  |  |  |  |  |  |

*indicates statistical significance
