# Supplementary material for: Multiple aspects of amyloid dynamics in vivo integrate to establish prion variant dominance in yeast
Source: Front Mol Neurosci. 2024 Jul 30;17:1439442. doi: 10.3389/fnmol.2024.1439442 (PMC11319303; doi:10.3389/fnmol.2024.1439442)
Supplement: Supplementary file 8 [file Table4.DOCX]

**Supplementary Table S4: p-values for [*PSI^+^*]^Weak^** **Propagons from Crosses in Figure 2B**

|  | [*PSI^+^*]^Weak^ haploid | [*PSI^+^*]^Strong^ X [*psi^-^*] | [*PSI^+^*]^Strong^ X [*PSI^+^*]^Strong^ | [*PSI^+^*]^Strong^ X [*PSI^+^*]^Weak^ | [*PSI^+^*]^Strong^ X [*PSI^+^*]^Weak^  (+GdnHCl) | [*PSI^+^*]^Strong^ titrated X [*PSI^+^*]^Weak^ |
| --- | --- | --- | --- | --- | --- | --- |
| [*PSI^+^*]^Weak^ haploid |  | 7.56E-06* | 5.77E-06* | 6.12E-03* | 2.06E-05* | 5.52E-03* |
| [*PSI^+^*]^Strong^ X [*psi^-^*] |  |  | 1.377E-01 | 3.24E-04* | 4.21E-04* | 2.79E-04* |
| [*PSI^+^*]^Strong^ X [*PSI^+^*]^Strong^ |  |  |  | 5.88E-04* | 2.10E-03* | 5.39E-04* |
| [*PSI^+^*]^Strong^ X [*PSI^+^*]^Weak^ |  |  |  |  | 2.046E-02 | 9.715E-01 |
| [*PSI^+^*]^Strong^ X [*PSI^+^*]^Weak^  (+GdnHCl) |  |  |  |  |  | 2.191E-02 |
| [*PSI^+^*]^Strong^ titrated X [*PSI^+^*]^Weak^ |  |  |  |  |  |  |

*indicates statistical significance
