# Supplementary material for: Multiple aspects of amyloid dynamics in vivo integrate to establish prion variant dominance in yeast
Source: Front Mol Neurosci. 2024 Jul 30;17:1439442. doi: 10.3389/fnmol.2024.1439442 (PMC11319303; doi:10.3389/fnmol.2024.1439442)
Supplement: Supplementary file 9 [file Table5.DOCX]

**Supplementary Table S5: p-values for [*PSI^+^*]^Weak^** **Propagons from Crosses in Figure 3**

|  | [*PSI^+^*]^Weak^ haploid | [*PSI^+^*]^Weak^ X [*psi^-^*] | [*PSI^+^*]^Weak^ X [*psi^-^*] (+GdnHCl) | [*PSI^+^*]^Weak^ X [*PSI^+^*]^Weak^ | [*PSI^+^*]^Weak^ X [*PSI^+^*]^Weak^ (+GdnHCl) | [*PSI^+^*]^Weak^ X [*PSI^+^*]^Strong^ | [*PSI^+^*]^Weak^ X [*PSI^+^*]^Strong^ (+GdnHCl) |
| --- | --- | --- | --- | --- | --- | --- | --- |
| [*PSI^+^*]^Weak^ haploid |  | 4.97E-07* | 1.122E-01 | 8.98E-08* | 1.38E-04* | 1.370E-01 | 1.65E-03* |
| [*PSI^+^*]^Weak^ X [*psi^-^*] |  |  | 1.55E-04* | 5.273E-01 | 1.74E-02* | 9.66E-08* | 4.24E-08* |
| [*PSI^+^*]^Weak^ X [*psi^-^*] (+GdnHCl) |  |  |  | 2.77E-04* | 3.607E-02 | 2.49E-02* | 4.07E-03* |
| [*PSI^+^*]^Weak^ X [*PSI^+^*]^Weak^ |  |  |  |  | 3.854E-02 | 1.12E-08* | 5.44E-09* |
| [*PSI^+^*]^Weak^ X [*PSI^+^*]^Weak^ (+GdnHCl) |  |  |  |  |  | 2.16E-05* | 5.45E-06* |
| [*PSI^+^*]^Weak^ X [*PSI^+^*]^Strong^ |  |  |  |  |  |  | 7.788E-02 |
| [*PSI^+^*]^Weak^ X [*PSI^+^*]^Strong^ (+GdnHCl) |  |  |  |  |  |  |  |

*indicates statistical significance
